# Healthy ageing at work— Efficacy of group interventions on the mental health of nurses aged 45 and older: Results of a randomised, controlled trial

**DOI:** 10.1371/journal.pone.0191000

**Published:** 2018-01-19

**Authors:** Imad Maatouk, Andreas Müller, Peter Angerer, Renate Schmook, Christoph Nikendei, Kirsten Herbst, Melanie Gantner, Wolfgang Herzog, Harald Gündel

**Affiliations:** 1 Department of General Internal and Psychosomatic Medicine, University of Heidelberg, Heidelberg, Germany; 2 Institute for Occupational Medicine and Social Medicine, Medical Faculty, University of Düsseldorf, Düsseldorf, Germany; 3 Department of Psychosomatic Medicine and Psychotherapy, University of Ulm, Ulm, Germany; TNO, NETHERLANDS

## Abstract

**Objective:**

This multicentre, randomised controlled trial (RCT) aimed to evaluate the efficacy of a small-group intervention promoting successful ageing at work in older nurses (aged ≥45).

**Method:**

A sample of 115 nurses aged ≥45 from 4 trial sites in Germany were randomly assigned to either the intervention group (IG), that received a small-group intervention of seven weekly sessions of 120 min with a booster session after six weeks or to a wait-list control condition (WLC). Outcomes were measured via validated self-report questionnaires at baseline (T1) and at post-treatment (T2).

Primary outcomes were mental health-related well-being and mental health-related quality of life (QOL). The secondary outcomes included mental health-related and work-related measures.

**Results:**

The intention to treat (ITT) analysis showed significant positive effects of the intervention on mental health. A significant small effect (d = 0.3) in favour of the IG was found for psychological health-related quality of life. Positive small effects (d = 0.24 to d = 0.31) were also found for work related mental strain.

**Conclusions:**

Our small-group intervention based on a theory of successful ageing for nurses aged ≥45 was found to be effective with regard to improvements of psychological health related quality of life and other mental health-related outcomes. Thus, our study shows that the ageing workforce can be reached through specifically designed preventive interventions. The components of our intervention could be easily adapted to the belongings of other professions. Our results suggest that these components should be evaluated in various settings outside the healthcare sector.

## Background

All European countries are facing demographic changes because of an increase in life expectancy and a decrease in fertility.[1, 2] The increasing proportion of elderly people is linked with respective care requirements and has profound consequences, such as high work volume and high emotional strain level, for workers in healthcare systems. According to the German Federal Statistical Office, changes will result in a shortage of more than 150,000 full-time nurses in Germany in 2025.[3] One possible strategy to cope with a nursing shortage would be to postpone the retirement age. However, only a few nurses want to keep practising until they reach the official retirement age due to the physical and social demands of the profession. Other important factors that lead to early retirement include work-related stress and impaired mental health.[4, 5] Compared to other professions, nurses frequently suffer from emotional distress or mental disorders.[6] According to results of the nurses´ early exit (NEXT)-Study, which had more than 28,000 participants from 10 European countries, a high psychological burden the strongest factor associated with an intention to leave (ITL) the profession. Comparisons among various age groups suggest a higher mental strain in older nurses than in their younger colleagues.[7] Therefore, effective preventive intervention programmes addressing the nursing staff workforce aged 45 and older are required to reduce work-related distress in order to maintain mental health until retirement age.

To the best of our knowledge, interventional studies that focus on the challenges of ageing workers are insufficient.[8] Regarding healthcare workers, there is no single efficacy study on interventions that focus on the special needs of nursing staff aged 45 and above.[9, 10] However, the process of successful ageing has been studied both theoretically and empirically. Gerontologists have been concerned with the study of theories of successful ageing during the last decades.[11] The most prominent construct—the Selection, Optimization and Compensation (SOC) model [12]—has been largely investigated in the context of occupational health.[13–16] Overall, the SOC model suggests that available resources can be used more efficiently by selecting fewer but carefully chosen goals (S), pursuing these goals optimally (O), and addressing barriers through compensatory means (C).[17] Exploratory research has shown that SOC is helpful in promoting employees’ psychological health across their work-life span[16]. Moreover, SOC can explain how older employees continue functioning despite the loss of resources.[18] In a recently published paper, Müller et al. demonstrated for the first time that training based on the principles of SOC could enhance the psychological well-being of nurses in a small sample with a mean age <45.[19]

Against this background, we primarily aimed to examine the efficacy of a small-group intervention for older nurses (aged ≥45) that combined elements of an occupational health intervention [20] based on SOC with several strategies for stress prevention (such as group dynamic principles and cognitive-behavioural techniques) in a multicentre, randomised, controlled trial. A framework proposed by the Medical Research Council for designing and evaluating complex interventions [21] was used to develop the intervention. In addition, the costs of the intervention were calculated.

## Methods

### Study centres and design

We conducted a two-armed randomised controlled trial (RCT) with two conditions, namely, a small-group intervention of seven weekly sessions of 120 min with a booster session after six weeks and a waiting list condition (WLC). The intervention group (IG) received the training, while the remaining participants formed the control group (WLC group), which received the training after the intervention group had finished the training.

The study was carried out at four trial sites in Germany. The medical hospitals of the University of Heidelberg, Düsseldorf, and Ulm as well as a community hospital in Aalen (county of Baden-Wuerttemberg) were the participating centres.

### Procedure and ethical approval

Participants were recruited in accordance with the staff council and the nursing management of all trial sites. Following the approval of the staff council and the nursing management, all nursing employees aged ≥45 were invited by email to participate in the study with the help of the respective human resources departments, which provided the lists of all relevant employees. In cooperation with the nursing directors, the staff council and the department heads, nurses were further intimated through information meetings, placards and written information about the intervention. The study purpose (Intervention to promote „healthy aging at work“) was explained in the informed consent and additional written materials as follows: “the aim is to explore your own resources, reflect your working biography, and improve personal resources and skills to maintain quality of life.”

It was agreed with the Staff Council that the survey and group training participation would take place during working time. All outcomes were measured via validated self-report questionnaires. No interview survey was performed. We have adhered to the ethical principles in accordance with the World Medical Association Declaration of Helsinki for Medical Research Involving Human Subjects of 2013. Written, informed consent was obtained from all the participants. Participation was voluntary, and participants could withdraw at any time without any consequences. The study was approved by the Ethics Committees of the Medical Faculties of the University of Ulm (ID: 358–11), the University of Düsseldorf (ID: 4537R; ID: 4675R), and the University of Heidelberg (ID: S-663/2013) in March 2014 (3-24-2014). The study was registered retrospectively in the ISRCTN registry (ISRCTN14793147). The authors confirm that all ongoing and related trials for this intervention are registered. In accordance with the study protocol that was approved by Ethics Committees before enrollment, the timetable (but not the content or dose) of the intervention was slightly modified. In a preliminary qualitative and a pilot study with the aim to test the intervention's feasibility and acceptance, participants advised an adaption of the manual from 10 sessions, 90 minutes a week and one booster session after 4 weeks (990 minutes) to a format with 7 sessions, 120 minutes a week and one booster session after 6 weeks (960 minutes). Details of the development and pilot process of the intervention have been published elsewhere [20]. In short, as the intervention was performed during working time, nursing staff needed a format with less appointments. A timetable with 7 sessions and one booster session after 6 weeks was more compatible with the rotating shift schedule of the participants. Ethical approval for the altered version of the timetable of the intervention was obtained in April 2014 (4-29-2014) before the study started. The Intervention started in June 2014. Further, the following deviations from the study protocol were made: Nursing staff criticised the length of the assessment. Three of the initially defined secondary outcome measures were rejected by the nursing staff and were therefore eliminated: the Maslach Burnout Inventory (MBI), the Nordic questionnaire, and the work related self-efficacy scale.

### Participants

We included currently employed nurses aged ≥45 who had sufficient skills in reading and writing German and had submitted written informed consent. Exclusion criteria were as follows: membership in a respective management team or leadership position, occupational disability (i.e. the continuing inability to continue working with patients due to physical or psychological impairment), cognitive impairment (i.e. not being able to follow the informed consent or other indications that the contents of the intervention cannot be followed) and serious physical or psychiatric illnesses at the time of recruitment. Symptoms of serious physical illness were defined as follows: untreated diseases requiring urgent diagnostics and further treatment such as severe shortness of breath, dizziness or angina pectoris. Severe psychiatric symptoms were defined as follows: requiring an immediate treatment such as acute suicidal tendencies, psychotic symptoms, dissociation or flash backs and severe addictive diseases.

### Randomisation procedure

Participating nurses were randomised to either the IG or the WLC group. Participants received a sealed envelope addressed to each personally and containing additional information about the study and the baseline questionnaire. To enable individual matching of the measurements, each participant was assigned digit codes to ensure pseudonymisation. Randomisation was performed after baseline assessment using a web-based randomisation program by an independent researcher who did not have any information about the participants. The intervention was not blinded as the participants were aware of their allocation to either the intervention or control groups.

### Intervention and control condition

Following comprehensive development (according to the guidelines of the Medical Research Council [21]), we conducted a two-armed RCT. The detailed procedure of intervention development has been published elsewhere [9, 20]. Small groups were led by one to two trainers (a psychologist and/or a doctor trained in psychotherapy and as a group leader). The minimum qualification was a degree in medicine or psychology and training or experience in psychotherapy/group leading with a working experience of at least two years. Approximately 10 employees comprised one group. The training included seven sessions, 2 hours a week, for a period of seven weeks. After a six-week break, there was another booster session. The WLC group received the same treatment after a break of one week after the IG’s booster session.

The key components of the single sessions have been described in detail in previous publications [9, 20]. Nevertheless, in the following section we would like to outline briefly the essential theoretical background and contents of the individual sessions:

#### Session one

**Introduction to the subject: „ageing in care professions”.** The first session had the aim to raise and to share consciousness of the mental health related risks of the nursing profession by giving information and discussing personal experiences. The first session also was developed to give the members of one group the possibility to create group cohesion. Apart from getting to know each other as a group, the discussion centered on what aging in care professions meant to participants, what difficulties they encountered and what personal resources they experienced. In the light of the health belief model, participants were encouraged to explore personal risk factors and resources (protective factors) to trigger internal cues of action. In terms of the trans-theoretical model of Behavior Change, we intended to reach the stage of contemplation, in which the participant’s awareness about the causes of high mental strain, that could be influenced by them increased.

#### Session two

**Reflecting the working biography.** Biography work was chosen to stimulate new self-understanding and, in turn, to awaken interest in the experiences of the other group members (in order to deepen the contents of the first group meeting). Group cohesion was strengthened. Against the background of the social cognitive theory [22] the session was created to share resources and experiences of overcoming barriers. It was hypothesized that an exchange with each other could strengthen personal self-efficacy and perceived collective efficacy. One key message (result) at the end of the session could be formulated as follows: “We have already overcome difficult situations during our careers and we are able to change our environment”. Exchange of working life experiences and the reframing of difficult memories may be an important intervention component.

#### Session three

**Coping with stress and the concept of mindfulness.** In terms of the transactional model of stress (Lazarus and Folkman), the intervention comprised several components of problem-focused and emotion-focused coping. [23, 24] Research has shown that employees can be taught skills reducing their stress levels and alleviating strain symptoms. During the third session, participants were taught emotional coping strategies such as mindfulness based stress reduction and other relaxation techniques. The mentioned strategies served as methods to improve emotional states. According to Bandura´s Social Cognitive Theory we hypothesized that participants accomplish a certain level of capability to improve emotional states in difficult situations.

#### Session six

In the sixth session, the focus was on reflecting age stereotypes and discussing the advantages and disadvantages of work between different generations including new perspectives on ageing.

#### SOC-focused sessions (four, five, seven, and booster session)

The SOC model was implemented in a problem focused way [19]. In terms of human action regulation, SOC can be defined as a combination of goal-related action strategies (goal setting theory [25]). In this action-theoretical formulation of SOC, the focus is on active strategies of life management. It is adaptive to set goals, acquire and invest means into the pursuit of these goals, and do so persistently even in the face of setbacks and losses. [17, 26] Therefore, the main component of the implementation of SOC within the intervention was to establish a personal SOC-project that aims toward more effective coping with individual job demands. Another aim was to activate an individually valued job resource. Participants define a specific goal (selection), develop an action plan to achieve this goal in an optimal way (optimization), and consider alternative strategies in cases of external or internal hindrances during goal accomplishment (compensation). Within the small group setting the participants worked together on setting goals and solving problems together in their peer group whenever barriers in achieving their goals occurred.

### Outcome parameter

All outcomes were measured via validated self-report questionnaires. All Questionnaires were self-administered by the participants during working time. No interview survey was performed. We assessed the participants at baseline (T1) and at post-treatment after a booster session after six weeks (T2). Demographic variables were assessed only at the baseline measurement.

### Primary outcome measures

We chose two *mental health-related* primary outcome measures: well-being and mental health-related quality of life (HRQOL).

#### Well-being

Well-being was measured by the World Health Organization (WHO)-5 questionnaire, a self-report measure comprising five items. This questionnaire has demonstrated good psychometric properties in several clinical samples of different age groups as well as in a large representative samples from the German population.[27] Research results have indicated that the WHO-5 is negatively associated with several measures for depression.[28]

The WHO-5 asks about the status of the last two weeks. Sample items of the WHO-5 are:

- `I woke up, feeling fresh and rested`
- `I have felt calm and relaxed`
- `My daily life has been filled with things that interest me`

The single items are rated on a 6-point Likert scale (from `all of the time`to `at no time`) High scores indicate an enhanced state of well-being. The scale has a high internal consistency with a Cronbach´s alpha of 0.92. [27]

#### Mental health related quality of life

We used the mental health subscale score of the World Health Organization Quality of Life-BREF (WHOQOL-BREF) to measure mental health related quality of life. The WHOQOL-BREF is a widely used abbreviated version of the WHOQOL-100 and contains a total of 26 questions.[29] Quality of life refers to an individual’s perceptions of several aspects of his/her position in his/her individual cultural context and personal value system. The concept of health-related quality of life (HRQOL) includes various domains that refer to the physical, mental (psychological) and social domains of health.[30] The WHOQOL-BREF produces scores that cover four different domains of HRQOL: physical health, mental health, social relationships and environment. Typical example items of the mental subscale are:

- `How much do you enjoy life?`
- `How satisfied are you with yourself?`
- `How well are you able to concentrate?`

The Items are each rated on a 5-point Likert scale. The respective subscale has a Cronbach´s alpha of 0.75. [29] The mental health score was calculated according to the manual. The importance of HRQOL as a relevant outcome variable in epidemiological and clinical studies is being increasingly recognised, and reduced HRQOL has been hypothesised as an independent marker of mortality.[31]

### Secondary outcome measures and additional variables

The secondary mental health-related outcomes were as follows: irritation, depressive symptoms and generalised anxiety symptoms.

#### Irritation, cognitive irritation, and emotional irritation

Perceived work-related mental strain was measured by the irritation scale. [32, 33] The questionnaire consists of eight items that are each rated on a 7-point Likert scale. High scores indicate high psychological strain. The scale is recommended in assessing psychological strain in occupational contexts. The three following scores can be calculated from the irritation scale: the overall score, 8–56 points; cognitive irritation, 3–21; and emotional irritation 5–35.

The concept of irritation can be considered a state of work-related mental strain that comprises two aspects reflected by the two subscales of irritation. First, cognitive irritation reflects the occurrence of thoughts about the problems at work (‘work specific rumination’). Sample items of the cognitive irritation subscale are:

- It's hard for me to switch off after work
- I have to think about difficulties at work at home too

The respective subscale has a Cronbach´s alpha of 0.87.[33]

Second, the concept of emotional irritation describes emotionally irritable reactions to work stressors. Sample items of the emotional irritation subscale are:

- When I come home from work really tired, I'm pretty nervous
- I react irritated, even though I don't want it at all.

The respective subscale has a Cronbach´s alpha of 0.88.[33]

Irritation is caused by the perceived non-attainment of work-related goals. Findings from longitudinal studies suggest that emotional irritation is a precursor of serious psychological impairment such as depression.[34]

#### Depression symptoms

The Patient Health Questionnaire-9 (PHQ-9) was used to assess the severity of depression symptoms. The proven psychometric properties of the PHQ-9 have been reported in various large-scale studies.[35]

The PHQ-9 scale inquires about symptoms in the past 2 weeks: `Over the last 2 weeks, how often have you been bothered by the following problems?`Sample items of the PHQ-9 are:

- `Little interest or pleasure in doing things`
- `Feeling down, depressed, or hopeless`
- `Feeling tired or having little energy`

Items are each rated on a 4-point Likert scale. Response options are `not at all`, `several days`, `more than half the days`, and `nearly every day`, scored as 0, 1, 2, and 3, respectively. The total score of the PHQ-9 ranges between 0 and 27 points. A cut-off point of ≥10 allows one to differentiate moderate to severe symptoms (10–27 points) from no (0–4) or mild depressive symptoms (5–9 points).The respective scale has a Cronbach´s alpha of 0.88. [36]

#### Generalized anxiety symptoms

The Generalized Anxiety Disorder Scale (GAD-7) was applied for assessing the generalised anxiety symptom severity. The GAD-7 is a validated instrument developed in a large primary care patient sample to assess anxiety symptom severity. [37]

The GAD-7 asks about recent symptoms (i.e., in the past 2 weeks): ‘Over the last 2 weeks, how often have you been bothered by the following problems?`

Sample items of the GAD-7 are:

- `Feeling nervous, anxious, or on edge`
- `Not being able to stop or control worrying`
- `Feeling afraid as if something awful might happen`

Items are each rated on a 4-point Likert scale. Response options are `not at all`, `several days`, `more than half the days`, and `nearly every day`, scored as 0, 1, 2, and 3, respectively. The total score of the GAD-7 ranges between 0 and 21. A cut-off point of ≥10 allows one to differentiate moderate to severe symptoms (10–21 points) from no (0–4) or mild anxiety symptoms (5–9 points). The German version of the GAD-7 has proved to have good psychometric properties in a large population-based sample. The respective scale has a Cronbach´s alpha of 0.89. [37]

#### Workability

The Workability Index Scale (WAI) was used to measure self-reported workability. It is the most popular measure for workability and has been used in numerous large-scale studies.[38] The definition of Workability is based on a so called ‘*concept of work ability’ of Ilmarinen*: Work ability can be described as *‘how good is the worker at present*, *in the near future*, *and how able is he/she to do his/her work with respect to the work demands*, *health and mental resources’*. [39] The questionnaire was developed to measure the level of work ability and to serve as a tool for investigating how long individuals are actually able to work.

In accordance with Müller et al.,[19] we applied three items of the WAI. The first item addressed current perceived workability compared to lifetime best work ability (rated on a scale from 0 = ‘completely unable to work’ to 10 = ‘workability at its best’):

- `Assume that your work ability at its best has a value of 10 points. How many points would you give your current work ability? (0 means that you currently cannot work at all)`

The second and third assessed items perceived current work ability related to physical and psychological job demands:

- `How do you rate your current work ability with respect to the physical demands of your work?`
- `How do you rate your current work ability with respect to the mental demands of your work?`

Items were rated on a 5-point Likert scale. We multiplied the value of the first item by 0.5 to calculate the sum score. The internal consistency of the instrument has been found to be good (Cronbachs alpha: 0.83). [40]

#### Job control

Working conditions in terms of job control were assessed by the nine items of the ´Work analysis instrument for hospitals-Self report version`(German: Das Tätigkeits- und Arbeitsanalyseverfahren für das Krankenhaus—Selbstbeobachtungsversion—TAA) questionnaire, a well-validated German self-report measure of job control [41] in hospitals. All items were rated on a 5-point Likert scale, regarding the degree to which their work allows for making own decisions.

Sample items of the TAA are:

- `I can decide for myself how I do my work`
- `I can set my own workflow`
- `I can implement my own ideas when carrying out the tasks`
- `I can be creative at the completion of tasks`

The respective scale has a Cronbach´s alpha of 0.77. [42]

#### Additional variables

The demographic variables comprised questions regarding work experience, sickness absenteeism and life circumstances, such as caregiving to relatives. Participants were asked the following questions:

`How old are you?`

`Are you male or female?`

`How many days of illness have you had in the last three months (12 weeks)?`

`Do you have children or relatives in need who live with you and/or you have to take care of`

`How long have you been working in nursing care (including training time; interruption times, e. g. due to parental leave etc. not taken into account)?`

`Do you work full-time or part-time?`

### Cost calculation

To calculate the costs of the intervention, several aspects were considered. The costs were calculated based on the salary scales for nurses, physicians and psychologists. The duration of preparing/performing the intervention and other expenses (taking into account the pilot groups, all travel costs, costs for the training of the trainers) were recorded. We performed two kinds of calculation: One considering the loss of working hours of nurses (based on final salary level) and one if the intervention was performed during leisure time. It must be emphasized that our intervention took place during working hours. No further calculations were made to record productivity losses. Cost of the group leaders was based on their respective salaries and estimated amount for time spent preparing and conducting group sessions. Working hours spent on piloting and development of the manual, discussions and the training day including all transportation costs and material (printing) costs were also considered.

### Statistical analyses

Our trial was powered to detect an effect size (according to Morris, 2008 [43]) of d = 0.5 (i.e. mean pre-post change in the intervention group minus the mean pre-post change in the control group divided by the pooled pre-test standard deviation of the main outcome measurement, see Morris) in the primary outcome (HRQOL) with an α of 0.05 and a power of 80% in a two-tailed test. The assumed difference between the groups was 5.0 with a pooled standard deviation of 10. Assuming a dropout rate of 10%, we required 144 participants in our study.

All analyses were performed according to the intent-to-treat principle. A conservative, single-imputation approach with ‘last observation carried forward’ (LOCF) was used to replace missing values at follow-up. In LOCF, missing follow-up data are replaced by the last observation. Therefore, in our study, the baseline score was used to replace the missing follow-up value of an individual, who did not provide follow-up data. For sensitivity analyses, per-protocol analyses with complete cases were conducted. Differences in the reported outcomes between the IG and WLC group were assessed using an analysis of covariance (ANCOVA) with the follow-up (T2)-score as the dependent variable, the baseline score as the covariate and group (IG or WLC group) as the predicting variable.[44] Effect sizes (d) were calculated in accordance with Morris [43]. Comparisons of different estimates favoured an effect size based on the mean pre/post change in the treatment group minus the mean pre/post change in the control group, divided by the pooled pre-test standard deviation. An effect size of d = 0.2 can be considered a small effect, d = 0.5 a medium and d = 0.8 a large effect. All analyses were performed using IBM-SPSS V24.

## Results

### Participants

Participants for the intervention were recruited from March 2014 until October 2014. The intervention was performed between June 2014 and July 2015. Outcome parameters were assessed with validated questionnaires at baseline and after the intervention.

**Fig 1:**
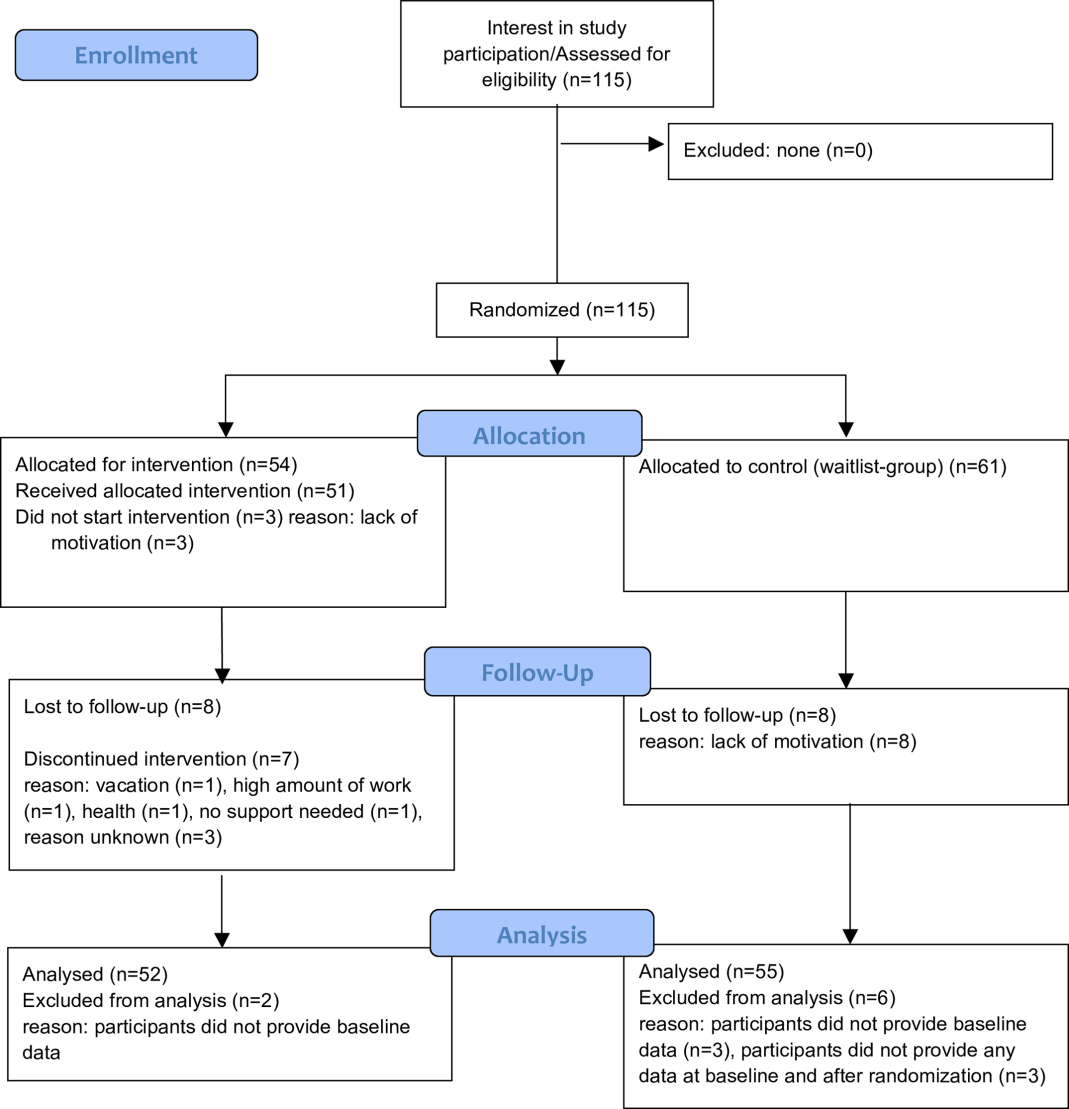
CONSORT 2010 flow diagram: Healthy ageing at work.

Altogether 115 registered German nurses (aged ≥45) working in three university hospitals and one community hospital in Germany participated in the study. The enrollment and flow of participants throughout the study is summarised in Fig 1. A total of 115 participants were randomly allocated to either the IG (n = 54) or the WLC group (n = 61). Study dropout rate was low (see Fig 1).

In total, 3 of the 54 participants allocated to the IG did not begin the intervention, and 2 did not provide baseline data, leading to their exclusion from analyses. In the WLC group, 6 participants were excluded, 3 for not providing baseline data and 3 for not providing any data before or after randomisation.

#### Baseline characteristics

The baseline characteristics of the participants are presented in Table 1. The mean age was 52 years with an average work experience of 30 years. Most of the participants were female. Table 2 shows descriptive data for all outcome variables at all assessment points.

**Table 1:** Baseline characteristics (T1) of participants of the intervention group (IG) and waiting list control (WLC) group.

Variable	IG	WLC Group
Age, mean (SD) in years	51.62 (4.65)	52.6 (5.56)
Gender, n (%)		
female	47 (87%)	45 (87%)
male	7 (13%)	7 (13%)
Work experience in nursing, mean (SD)in years	28.83 (6.36)	30.36 (7.84)
Mode of employment		
Full-time, n (%)	17 (31.5%)	24 (47%)
Part-time, n (%)	37 (68.5%)	27 (53%)
Reported caregiving for relatives, n (%)	20 (37%)	24 (46%)
Sickness absenteeism, mean (SD) in days (relating to the last 3 months)	3.15 (4.09)	2.52 (4.2)

SD = standard deviation.

**Table 2:** Means and SD of outcome variables and baseline (T1) and follow-up (T2) (IG and WLC).

	T1				T2			
Group	IG		WLC Group		IG		WLC Group	
Outcome	Mean	SD	Mean	SD	Mean	SD	Mean	SD
Well-being (WHO-5)	11.68	5.31	12.76	4.12	13.57	4.71	13.84	4.79
Mental health- related quality of life (WHOQOL-BREF)	65.56	14.99	68.3	12.37	68.95	12.83	67.5	13.86
Irritation (Irritation scale)	27.39	9.68	27.84	9.78	23.49	9.29	26.98	9.65
Cognitive irritation	11.86	4.39	12.14	4.4	10.49	4.21	11.90	4.22
Emotional irritation	15.45	6.27	15.98	6.51	12.99	6.25	15.08	6.79
Depression Symptoms (PHQ-9)	8.04	4.44	6.97	3.98	6.92	4.38	6.67	3.83
Generalized Anxiety Symptoms (GAD-7)	6.42	4.04	5.5	3.87	5.43	3.37	5.04	3.43
Workability (WAI)	6.34	1.44	6.99	1.51	6.98	1.35	7.16	1.42
Job Control (TAA)	3.08	0.62	3.08	0.75	3.21	0.64	3.17	0.75

IG = intervention group; WLC = waitlist condition; SD = standard deviation

WHOQOL-BREF = World Health Organization Quality of Life-BREF; PHQ-9 = Patient Health Questionnaire-9; GAD-7 = Generalized Anxiety Disorder Scale-7; WAI = Workability Index Scale; TAA = Work analysis instrument for hospitals.

### Effectiveness of the study

#### Primary outcome measures

The intention to treat (ITT) analysis showed significant positive effects of the intervention with regard to mental health. A significant small effect (d = 0.3) in favour of the IG was found for psychological health-related quality of life. No significant difference between the IG and WLC for well-being was detected (Table 3).

**Table 3:** Results of the ANCOVA for primary and secondary outcomes.

	Between groups effects T2		
		Results of ANCOVA	
Measure	Effect size d_ppc2_(Morris et al. [35])	F	p-value
Mental health-related quality of life	**0.3**	**4.122**	**0.045**
Well-being (WHO-5)	0.17	0.036	0.851
Irritation (Irritation scale)	**0.31**	**8.76**	**0.004**
Cognitive irritation	**0.26**	**9.08**	**0.003**
Emotional irritation	**0.24**	**4.34**	**0.04**
Depressive Symptoms (PHQ-9)	0.19	0.98	0.326
Generalized Anxiety Symptoms (GAD-7)	0.13	0.169	0.681
Workability (WAI)	0.32	1.57	0.213
Job Control (TAA)	0.06	0.32	0.537

Missing values were replaced by single imputation (`last observation carried forward’).

ANCOVA = analysis of covariance; SD = standard deviation; PHQ-9 = Patient Health Questionnaire-9; GAD-7 = Generalized Anxiety Disorder Scale; WAI = Workability Index Scale; TAA = Work analysis instrument for hospitals; T2 = follow-up.

#### Secondary outcome measures

Positive effects were also found for the following mental health-related outcome measures: overall irritation, emotional irritation and cognitive irritation. The effect sizes were small and ranged from d = 0.24 to d = 0.31.

No between-group effect for depressive symptoms or generalised anxiety symptoms was found. Further, no significant differences between IG and WLC were found for work-related outcome measures.

#### Sensitivity analyses

The results of the Per Protocol (Complete-Case) analysis were close to the ITT results and revealed similar effects for mental health-related outcomes. However, a small but significant difference for workability was found in favour of the IG.

#### Costs of the intervention

Considering the loss of earnings (intervention during working time), the intervention costs approximately €436 per participant. If the intervention is performed during leisure time, costs amount to €109 per participant.

## Discussion

This is the first study to evaluate the efficacy of a small-group intervention that focused on the promotion of mental health for nursing staff aged ≥45 in a randomised controlled study. Despite the aforementioned challenges of an ageing workforce in nursing with a high ITL the job because of mental strain, almost no intervention has focused on the specific requirements of ageing nurses. Our study shows that it is possible to achieve participation of ageing workers by using approaches that are tailor-made for the specific needs of that population. The results of our work show statistically significant small effects on different mental health-related outcome measures such as mental health-related quality of life and irritation. No significant differences between groups were found for well-being, depression and anxiety symptoms or work-related outcome measures such as the WAI and TAA.

### Prevention in healthcare workers

The reported results support the effectiveness of our intervention in reducing work-related mental strain and improving mental HRQOL. The reported effect sizes are comparable to those from other studies in the health care sector not focusing on our target population of ageing workers [10]. No effect of our intervention was found on well-being. Well-being that was measured with the WHO-5 Well-being Index has strong negative associations with depression measures. The items of the WHO-5 comprise three core symptoms of depression, such as ‘(lack of) positive mood, interests and energy’.[28] The lack of statistically significant differences between the IG and the WLC group regarding well-being could be due to a ceiling effect. Compared to a clinical sample, the participants of our study did not have a high mental health burden. Therefore, we were unable to generate significant changes in the well-being scores through our intervention.

However, the levels of irritation were significantly reduced through our intervention. At baseline, the mean levels of irritation were higher than the norm sample (n>4000) from working and general populations [33, 45]. This corresponds to the findings of many studies that have indicated a higher risk for mental strain and impaired mental health in nurses than that in the general population. Furthermore, this finding is in line with data that suggests a higher mental strain in the older compared to the younger workforce [32]. No effects on anxiety and depression symptom severity were detected. This finding could be a result of moderately elevated depression and anxiety symptoms before the start of the intervention; thus, it could also be explained by a ceiling or a floor effect. Another explanation could be that the clinical outcomes of depression and anxiety are less sensitive to change in the context of our preventive intervention.

With regard to healthcare professionals, several individual training and organisational intervention approaches aimed at preventing or reducing work-related stress have been investigated in RCTs [46–50]. However, in a recently published Cochrane review, the authors rated the quality of the evidence of general preventive interventions in healthcare workers as low because of a lack of well-designed randomised controlled trials. For example, in comparison to our study, power calculations were often missing, or studies were not registered [10]. Furthermore, many of these studies faced high attrition and had only few participants.

### Selective vs. indicative approaches

Promoting health in the workplace is the most important part of prevention. Prevention programs offered at the workplace provide a good opportunity to reach people at high risk for mental strain. Preventive interventions can be administered to individuals at high risk (selective prevention) or to individuals with subclinical symptoms (indicated prevention).[51] Contrary to indicative prevention studies,[50, 52, 53] we did not choose a high level of mental strain as an inclusion criterion. Instead, we invited all employees aged ≥45 interested in participating. A major advantage of our selective (instead of an indicative) approach is that stigmatisation due to mental health-related problems was precluded. From a scientific perspective, the major disadvantage of a selective approach is the loss of statistical power to detect changes in clinical outcome measures.[51] However, stigmatising attitudes towards employees with mental disorders seem to be a major reason for the delay in seeking help and could serve as an explanation for the attrition reported in other studies.

### Preventive interventions for the ageing workforce

Even regarding other occupations, there is little evidence on interventions targeting the successful ageing of the working population.[54] In a recently published review, Cloostermans et al. noted a dearth of studies in the area of an ageing workforce.[8] Compared to our study, the researchers chose a relatively low cut-off age of 40 years and could only identify four studies that met their inclusion criteria. Moreover, of the four studies, only two specifically targeted ageing workers (≥40 years), while the other two studies performed subgroup analyses of participants aged ≥40. It can be concluded that there is a paucity of interventional studies with regard to the ageing workforce. What could be the reason for this? It must be noted that not only mental health-related issues but also the focus on an ageing workforce is possibly stigmatising and could prevent employees from participating in (age-) specific interventions. Negative (deficit-oriented) age stereotypes are widespread in western societies [55, 56] and could also be a reason for the lack of interventional research on this important topic. Theoretical and empirical findings from gerontology, biological science, mental and social aspects of ageing, could serve as an inspiring source for intervention development as shown by the present study and the ´Alternsgerechte Pflegearbeit´ (ALPA) study conducted by Müller et al.[19].

### Theories of successful ageing

Gerontologists have formulate a several theories for successfully dealing with ageing.[11] Our study shows that these theories can be successfully transferred to the practical field of occupational health prevention, focusing particularly on older nurses. In our study, ageing was considered in the context of a popular theory of successful ageing: SOC, a strength-based (instead of a deficit-based) approach enabling a positive, resource-oriented view on the process of ageing. [12] Research has shown that SOC at work is a useful strategy for maintaining mental health across the work-life span[16]. The ALPA study conducted by Müller et al. already demonstrated that an intervention based on SOC could increase nurses’ well-being. However, compared to our work, the study did not provide sufficient means to detect statistically significant changes.[19] In addition, compared to our work, the mean age of participants was below 45 years, and the study was conducted in a single community hospital with a smaller sample. In our study, we included some components of the ALPA study and performed a multicentre trial that led to a large sample of >100 participants.

### Strengths and limitations

A major strength of our study is its design of a randomised controlled trial, based on a comprehensive developmental process already published [20]. A detailed description of the components of the intervention has also been made available through publication. In cooperation with researchers/clinicians from occupational medicine, psychosomatic medicine and occupational and organisational psychology, we performed a multicentre study that was successfully applied at three different academic and one community hospitals. Further, we used several work-related and clinical outcome measures. Most prior studies did not account for the important outcome variable of HRQOL.[10] Quality of life has become an important outcome in clinical studies and should also matter to occupational health researchers: ‘Occupational medical research has been fairly late in coming to consider quality of life as a focus of investigation’.[57]

However, our study has certain limitations that should be considered while interpreting the results. First, we could not reach the expected sample size. Therefore, our study could be limited and could therefore have failed to detect significant effects on well-being and other mental health-related variables, such as anxiety and depression. In a future study, the RCT should be replicated in a larger project undertaking a larger number of experiment centres, including community hospitals.

Second, as our intervention consisted of several components that have been described in detail elsewhere, it seems difficult to identify the single components and reasons responsible for the observed effects of our intervention. However, from a clinical and a health services research perspective, we are convinced of the combination of multiple components in the prevention of mental health.

A noteworthy limitation of our study is that some nurses suffering from very high mental strain or even depression (those with the strongest need for intervention) maybe could not be reached by voluntary participation. However, employees with more severe psychological burden should be placed in regular treatment such as psychotherapy or a combination of psychotherapy and drug therapy after careful examination and diagnosis. We had to offer the intervention to all nurses aged 45 and older to prevent any discriminatory practice. However, our study compares favorably with many others who did not recruit as many nurses as we did. It should also be emphasized, that we could not exclude spill-over effects, since participants were working in the same hospital. However, the risk of contamination is estimated to be very low in a three-shift system with a high workload and a large number of employees. Further, if it had occurred it would have induced a negative bias, resulting in an underestimation of a true intervention effect.

Another limitation is that the long-term effects of the intervention remain inconclusive. For example, a relevant and interesting outcome that can be effectively addressed by preventive interventions in an older adult workforce is the reduction of early retirement. To deal with this important issue, further studies with long-term follow-up measurements in the health care sector are required.

### Conclusion

In conclusion, this randomised controlled multicentre study indicated that participants in a small-group intervention based on a theory of successful ageing for nurses aged ≥45 showed significant improvements in several mental health-related outcomes. Thus, our study shows that the ageing workforce can be reached through specifically designed preventive interventions. Future studies have to examine if the components of our intervention can be easily adapted to the belongings of other professions. Our results suggest that these components should be evaluated in various settings outside the healthcare sector.

For this purpose, our study may provide a possible blueprint for future occupational interventions addressing the consequences of demography.

## Supporting information

S1 File Development paper. (PDF)

S2 File Study protocol–English. (PDF)

S3 File Study protocol original–German. (PDF)

S4 File Consort checklist. (DOC)

S1 Table Variables. (XLSX)

S2 Table Data with replaced missing values. (XLSX)

S3 Table Data with missing values. (XLSX)
