# Supplementary material for: Healthy ageing at work— Efficacy of group interventions on the mental health of nurses aged 45 and older: Results of a randomised, controlled trial
Source: PLoS One. 2018 Jan 19;13(1):e0191000. doi: 10.1371/journal.pone.0191000 (PMC5774702; doi:10.1371/journal.pone.0191000)
Supplement: S1 File — (PDF) [file pone.0191000.s001.pdf]

Mental Health & Prevention 4 (2016) 42–48

Contents lists available at ScienceDirect

Mental Health & Prevention

journal homepage: www.elsevier.com/locate/mhp

Healthy aging at work – Development of a preventive group intervention to promote quality of life of nursing staff aged 45 years and older

I. Maatouk [a,*], A. Mueller [b], R. Schmook [b], P. Angerer [b], K. Herbst [a], A. Cranz [a], E. Voss [d], H.J. Salize [d], M. Gantner [c], W. Herzog [a], Harald Gündel [c]

[a] Department of General Internal and Psychosomatic Medicine, University of Heidelberg, Germany
[b] Institute for Occupational Medicine and Social Medicine, Medical Faculty, University of Düsseldorf, Germany
[c] Department of Psychosomatic Medicine and Psychotherapy, University of Ulm, Germany
[d] Mental Health Services Research Unit, Central Institute of Mental Health, Mannheim, Germany

ARTICLE INFO

Article history:
Received 13 November 2015
Received in revised form
11 January 2016
Accepted 19 January 2016
Available online 26 January 2016

Keywords:
Prevention
Stress prevention
Older workers
Qualitative research methods
SOC

ABSTRACT

Background: Demographic change is challenging health care. In Europe, shortfalls in nursing numbers already exist due to low retention rates. The development of prevention programs addressing nursing staff aged 45 and older and aiming to enable older nursing staff to remain healthy until retirement age is needed.
Objective: The development and evaluation of a complex prevention program for the target group of nursing staff aged 45 and older incorporating key elements of occupational health interventions.
Methods: We focused on nursing staff aged 45 and older and used a framework that is proposed by the Medical Research Council (MRC) for the design and evaluation of complex interventions. Prior to the definitive RCT regarding effectiveness, we aimed to explore the main sources of workplace distress and possible prevention. Additionally, a qualitative study with nursing managers and the ward management and experiences of a pilot study informed about barriers and enablers of the intervention implementation.
Results: In this article, we provide a detailed description of the developmental process and the final intervention manual that will be evaluated in a RCT.
Conclusion: A complex intervention program can be implemented for nursing staff aged 45, reach the targeted population, and gains high acceptance. Further research on feasibility and effectiveness is indicated.
© 2016 Elsevier GmbH. All rights reserved.

1. Introduction

In most European countries, shortfalls in nursing numbers already exist due to low retention rates. However, demographic change has led to profound consequences for health care. According to current projections the number of long-term care patients in Germany may increase from currently 2.25 million to 2.9 to 3.3 million patients by the year 2030. Over the last years, working conditions have changed (e.g. increased need for care, patients with multimorbidity, time pressure, and documentation obligations) leading to an increase of work strain, work related stress, and job dissatisfaction (Isfort et al., 2010). Job dissatisfaction is a strong predictor for the intent to leave nursing (Coomber & Barriball, 2007). According to statistics by the German Federal Statistical Office, these changes will entail a shortage of more than 112.000 full time nursing staff within the next 10 years (Afentakis & Maier, 2010). Hence, to cope with these problems, there is a need to develop prevention programs addressing nursing staff aged 45 and older and aiming to enable older nursing staff to remain healthy until retirement age. There is strong evidence that nurses in Germany that are 45 years and older report meaningfully reduced physical and psychological capabilities to perform their job (Camerino et al., 2006). Thus, nurses at this age seem to be highly sensitive to age-related changes and an exemplary professional group to address questions prevention programs that aim to improve healthy aging at work.

In the last decades the effectiveness of general preventive strategies for dealing with work related stress has been examined in randomized controlled designs. Most strategies, such as cognitive-behavioral or relaxation techniques, focus on individual coping. In addition, organizational interventions focus on the development of the individuals' working environment (Richardson & Rothstein, 2008). However, to our knowledge, only few studies

* Correspondence to: Department of General Internal and Psychosomatic Medicine, University of Heidelberg Medical Hospital, Thibautstrasse 2, 69115 Heidelberg, Germany.
E-mail address: imad.maatouk@med.uni-heidelberg.de (I. Maatouk).

http://dx.doi.org/10.1016/j.mhp.2016.01.002
2212-6570/© 2016 Elsevier GmbH. All rights reserved.

have focused on the special needs nursing staff aged 45 and older have and the challenges they face.

Müller et al. introduced one of the most prominent models of successful ageing: selection, optimization, and compensation (SOC) (Baltes & Baltes, 1990) in nursing. As a model of long-term individual life management, (S) selection involves the prioritization of goals or activities to maintain or improve abilities, with (O) optimization strategies helping to identify and utilize resources in a goal-directed manner and compensation, similarly, involving the modification of activities or the use of adaptive aids with the aim of minimizing the impact of depleted goal-related means (Baltes & Baltes, 1990). In general, the SOC model proposes that individuals manage potential age-related losses in resources more efficiently by virtue of those three interrelated action strategies, i.e. focussing on fewer, but important goals, pursue these goals in an optimized manner, and, in doing so flexibly apply adequate compensatory means to address goal-relevant barriers (Baltes, 1997). In a large cross-sectional study, researchers have shown a significant association between the use of SOC-strategies and high work ability of nurses (Muller, Weigl, Heiden, Glaser, & Angerer, 2012; Müller et al., 2013). Thus, SOC strategies in nursing seem to be associated with criteria of successful aging at work (Zacher & Frese, 2011). Against this background, Müller et al. developed a training program on the basis of the SOC theory with the aim of enabling nurses to develop more effective work-related stress coping strategies as well as changing working conditions in a personal SOC-project. However, the considerable number of participants who discontinuing the program because of overburdening personal projects, which were difficult to implement, proved to be a critical issue of this SOC-training program (Müller, Heiden, Herbig, Poppe, & Angerer, 2015). The authors concluded, that future SOC-trainings should also entail other established components of occupational health interventions (e.g. relaxation techniques) to enable more flexible stress management that better fits the specific needs of participants.

Therefore, our aim was to develop and to evaluate a complex prevention program combining key elements of occupational health interventions guided by theory and evidence. In the present paper, we aim to describe the development process of a complex occupational health intervention for nursing staff aged 45 and older. The intervention's focus lies on enabling nurses to remain healthy until retirement age. A framework proposed by the Medical Research Council (MRC) for the design and evaluation of complex interventions (Campbell et al., 2000) was used for the development.

2. Material and methods

First, we adopted the theory and modeling phase recommended by the U.K. Medical Research Council (MRC) for the investigation of complex interventions (see Fig. 1).

After a literature review (Phase 0), we developed an interview questionnaire and implemented a needs assessment to gain an inside view of the problems and difficulties encountered by older nursing staff (Phase 1). Based on theory as well as our experience from interviews on causes for increasing stress levels in nursing, we developed a small group prevention program for older employees in nursing (Phase 2). In a next step, we ran a pilot group to gain practical knowledge of our intervention (Phase 3). The process and results of the development are described below.

2.1. *Phase 0, theory: research of literature, existing programs, and topics*

First, in order to identify the main difficulties of aging and work ability in hospital nursing, we conducted a literature review concerning job stress components, changes in nursing environments, increasing job requirements and useful theories of aging. Research on workplace stressors in nursing is extensive. In a comprehensive review, McVicar identified six main sources of workplace distress in nursing (McVicar, 2003): (1) high workload with insufficient staff cover and time pressure, (2) poor staff relationships (3) poor leadership and management style including the lack of supervisory support, (4) occupational nursing requirements including coping with emotional needs of patients and their families and poor patient outcomes, including death, (6) shift work considered to be a risk factor for many health-related problems, such as diabetes (Gan et al., 2014), and finally (6) a lack of effort reward, such as adequate income, possibilities for personal development, recognition and respect within a hierarchical system (Hasselhorn, Tackenberg, & Peter, 2004). Accordingly, these factors result in an ample amount of difficult work-life experiences over time.

Second, we compared different existing stress prevention strategies reported to have had an empirically measurable impact on mental and physical health. During research, we aimed at answering the following questions: (1) is there evidence for the effectiveness of stress prevention at work place? Which interventions are already known to be effective in stress prevention? (2) Are there specific interventions for healthcare workers? Which concept seems to be useful in the healthcare sector? (3) Is there evidence regarding interventions in older workers?

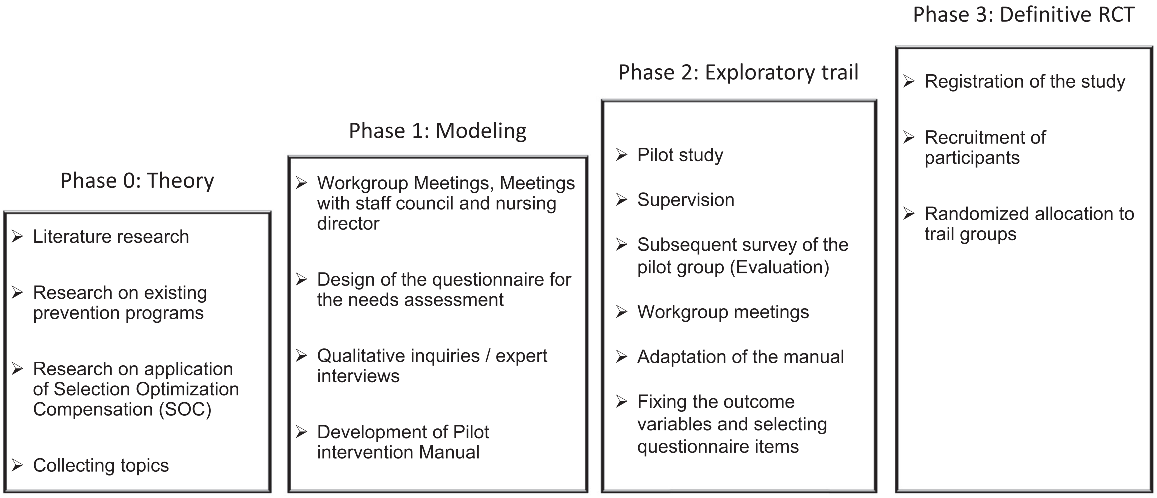

Fig. 1. Phases of intervention and study development corresponding to the framework proposed by the medical research council (MRC).

In regard to our first question, a meta-analysis by Richardson and Rothstein, aiming to determine the effectiveness of stress management interventions in occupational settings, included 36 experimental studies representing 55 interventions (Richardson & Rothstein, 2008). Interventions consisted of cognitive behavioral, relaxation, organizational, multimodal, or other components with cognitive behavioral programs producing larger effects than others. Furthermore, while many studies included relaxation interventions, organizational aspects were hardly taken into account. With an average intervention length of 7 weeks, the authors found a significant medium to large short-term effect of stress management interventions in occupational settings. Overall, little is known about the long-term effects of stress management interventions. However, Limm et al. found evidence that a stress management intervention (SMI), based on the effort-reward imbalance model of work-related stress and using modified techniques of group psychotherapy, reduces perceived stress reactivity and biological stress indices after 1 year of follow-up (Limm et al., 2011).

Concerning our second question, in 2014, a Cochrane review on the prevention of stress in healthcare workers was performed by Ruotsalainen et al. Taken together, researchers found evidence that several interventions, such as cognitive-behavioral or relaxation techniques, reduce healthcare workers' work-related stress symptoms levels. However, the quality of evidence was low, e.g. power calculations were often missing and studies were often only performed in a single department (Ruotsalainen, Verbeek, Marine, & Serra, 2015).

In light of our third question, a recently published systematic review found limited, due to few RCTs and inconsistent findings, evidence for a favorable effect of interventions promoting work-related components in older workers. Hence, further research is needed to provide evidence for the effectiveness of preventive interventions in older workers. Alarmingly, there was no study that focused on the needs of older workers in the healthcare sector (Cloostermans, Bekkers, Uiters, & Proper, 2014).

2.2. *Choosing elements for the design of the intervention*

Based on our own clinical experience and the literature research, we defined several important elements for the design of our intervention:

2.3. *Group treatment*

Group treatments seem to show several advantages compared to individual settings (Lambert, 2013). First, when cost-effectiveness is included in equations, group settings are less costly than individual treatment. Moreover, several key factors appear to be related to positive outcomes in group settings: apart from the participants' experience of similar problems faced by others, a positive group climate, as well as a feeling of mutual understanding within the group are beneficial aspects of group relationships formed. In addition, the construct of the development of a sense of group belonging (group cohesion) has been well investigated. Studies have shown group cohesion to be related to a positive treatment outcome (Johnson, Burlingame, Olsen, Davies, & Gleave, 2005). Hence, in light of the described economic and therapeutic advantages, we chose the therapeutic concept of small group treatments for our program.

2.4. *Individual stressors and coping with stress*

Stress management trainings, in which participants successfully learn to achieve flexibility in coping with daily stresses, have been proven to be beneficial for mental and physical health. Research has shown that individual employees can be taught techniques reducing their stress levels and alleviating strain symptoms (Richardson & Rothstein, 2008). In line with a popular, multimodal, stress management training program (Kaluza, 2012), we decided to include a session identifying participants' individual personal stressors and coping strategies.

2.5. *Biographical work*

Nurses in an advanced stage of their working career may have faced many difficult experiences. Research on psychotherapy in older adults indicates that life review techniques, looking back and acknowledging positive working life achievements, may be an effective intervention for dealing with the past (Haber, 2006). Therefore, in the complex undertaking to improve mental health, the exchange of positive and negative working life experiences and the reframing or reinterpretation of difficult memories may be an important intervention component.

2.6. *A useful theory of successful aging: selection, optimization, compensation*

In the past decades, the conception of successful aging has been an important subject of gerontological research. One of the most prominent theories of successful aging is the model of selection, optimization, and compensation. As a universal model of change adaptation occurring in older age, the SOC model has been useful for the explanation of the adaptive allocation of individual resources. In regard to coping with age-related problems, the model provides a valuable theoretical framework. It assumes that an individual's available resources can be used more efficiently when one focuses on fewer personal motives (selection) and optimizes goal attainment by either substituting losses or maintaining a certain level of functioning (compensation). Research shows that SOC is useful in promoting employees' mental health across the entire work-lifespan (Müller & Weigl, 2015). Müller et al. combined the SOC model's action-theoretical approach (Freund & Baltes, 2000) with work design as proposed by the Job-Demands-Resources Model (Demerouti, Bakker, Nachreiner, & Schaufeli, 2001). A main component of SOC training was the establishment of a personal project for each participant aiming toward more effective job strain coping in nursing (Müller et al., 2015). In a first step, each participant chooses a specific goal (selection) and then develops an optimal goal attainment plan (optimization) in a second step. Third, alternative strategies in case of external or internal obstacles during goal attainment (i.e., compensation) are considered. This process is supported through the discussion of individual projects within a small group setting. We aimed at adopting several steps from the SOC training and at including them in our training program.

2.7. *Age stereotypes*

In regard to older employees, age stereotypes seem to be significantly associated with well-being and health. In a recent study, a research group from the Yale School of Public Health has shown an increase of negativity of age stereotypes in western societies during the last 200 years (Ng, Allore, Trentalange, Monin, & Levy, 2015). Stereotypical beliefs about aging may negatively impact the self-view and wellbeing of older workers (Rothermund & Brandtstädter, 2003). An intervention seeking to improve older employees' well-being should therefore take age stereotypes into account in order to reduce the negativity of participants' own representations of aging.

2.8. Relaxation techniques

Relaxation techniques are frequently used in stress prevention interventions. Relaxation aims at enabling employees to reduce adverse reactions to stress by achieving a physical and/or mental state that is the physiological opposite of stress (Richardson & Rothstein, 2008). Taking evidence for several relaxation techniques into account, we decided to choose a compilation of methods: (1) autogenous training (Schultz & Luthe, 1959), (2) progressive relaxation training (Carlson & Hoyle, 1993), (3) mindfulness based stress reduction exercises (Grossman, Niemann, Schmidt, & Walach, 2004), imagination exercises (Reddemann, 2010).

3. Phase 1: modeling

3.1. Qualitative study

As a next step, we developed a semi structured questionnaire for a qualitative needs assessment. We wanted to elucidate possible preventive intervention promoters and obstacles. In addition, we aimed to gather information on individual/organizational resources which could be helpful for the establishment of personal projects. Furthermore, we wanted to identify main workplace distress sources. The questionnaire was developed without fixed expectations on specific work-related stress manifestations in nursing.

We used qualitative interviews with the nursing manager and the ward management to gain a deeper understanding of problems and topic-related themes. We were particularly interested in the employees' views on the changes in nursing work over the past years, perceived main stress components, as well as differences between younger and older nursing staff and resulting potential conflicts.

Furthermore, we wanted to elucidate emerging issues, specific worries and stresses, as well as useful resources and solutions already practiced or suggestions for improvement. In a general ward manager meeting, information on the planned prevention program was given and nursing and ward managers of the University of Heidelberg, Medical Hospital, invited to participate in a voluntary needs assessment interview. Interviews were conducted by a trained psychologist in 5 different focus groups with 5 participants each. Additionally, nursing managers were individually interviewed at two centers (Heidelberg, Ulm). Data were collected using semi-structured interviews (Flick, Kardorff, & Steinke, 2010) and focus groups (Bohnsack & Przyborski, 2006) with the same interview guidelines being used for individual and focus group interviews. However, some topics were discussed more intensively within the focus groups. As their opinion of their individual problems was of special interest, adequate room for individual point of views and statements was allowed, although ward and nursing managers shared similar themes. The interview data from interviews and focus groups were analyzed separately by using MaxQDA (2010 version, VERBI GmbH, Berlin) and examined thematically (Mayring, 2008). Key topics and themes were identified and reanalyzed within the theoretical framework and conclusions concerning the prevention program were made. Key topics were in line with our literature review but complemented by center-specific issues. The results of the qualitative study will be published elsewhere.

3.2. Development of the manual

On the basis of the extensive literature review, and after data analysis of the transcribed interviews as well as discussion of the results with an interdisciplinary team (medical doctors, psychologists), we started to plan the intervention's single sessions and chronological sequence. Results of meetings with staff council and the nursing directors were also taken into account. Based on group dynamic principles, we chose a small group setting with maximum 10 participants. Along with the results of Phase 0 and Phase 1, the intervention consisted of the following components: (1) identification of individual stressors and modification of personal strategies to cope with stress, (2) biographical work (focused on working life), (3) SOC training in line with Müller et al.'s intervention. Each participant was required to establish a personal project aiming toward more effective coping with a relevant important job demand or to activate a relevant, valued work-related resource. (4) Age stereotype work. Participants were invited to discuss own and general views on aging as well as to gain Information on the possible negative effect of age stereotypes on self-view and work performance. However, positive aspects of aging at work were emphasized. In addition, potential conflicts in heterogonous teams (with younger and more experienced employees) were discussed. (5) Relaxation exercises (as mentioned above) were selected on existing evidence and one planned to be implemented at the end of every session for approximately 20 min. In addition, a professionally produced stress-prevention CD with detailed instructions of the mentioned relaxation exercises was given to all participants. The participants were encouraged to practice the exercises at home.

4. Phase 2: exploratory trial and evaluation process

In the next phase, the collected information was used to develop an optimal intervention program. Our main aim was to test the intervention's feasibility and acceptance by participants. After initial development, we tested the implementation of the program with a pilot group of nine volunteers. The pilot intervention consisted of ten weekly sessions lasting 90 min each. Following the pilot intervention, we evaluated our experiences and adapted the manual for the main trial. Additionally, current developments in research were taken into account. Resulting intervention manual modifications used for this evaluation study will be described in the results section, while relevant pilot trial results are discussed below.

Overall acceptance for the intervention was high. The biographical session promoted closeness and led to a better mutual understanding and feeling of empathy. The SOC sessions turned out to be more time consuming than expected. It took participants a while to identify suitable projects and to outline ideas for implementing them in their work routines. Furthermore, group work on age stereotypes resulted in a lively discussion including self-reflection and new perspectives on ageing. One of the most important observation made, was the pronounced impact of group dynamic principles (sharing problems and discussing it with each other) leading to strong group cohesion.

Following the intervention, every participant was interviewed individually and asked for feedback concerning the sessions' usefulness, beneficial methods, theories, the most effective tools and advice, information gain, reflections and critical aspects of the intervention.

5. Results

5.1. Final intervention after manual adaptation

Thematically, every session focused on a different topic (see Fig. 2). Every session was ended with a 10–20 min relaxation exercise and participants were also encouraged to practice these

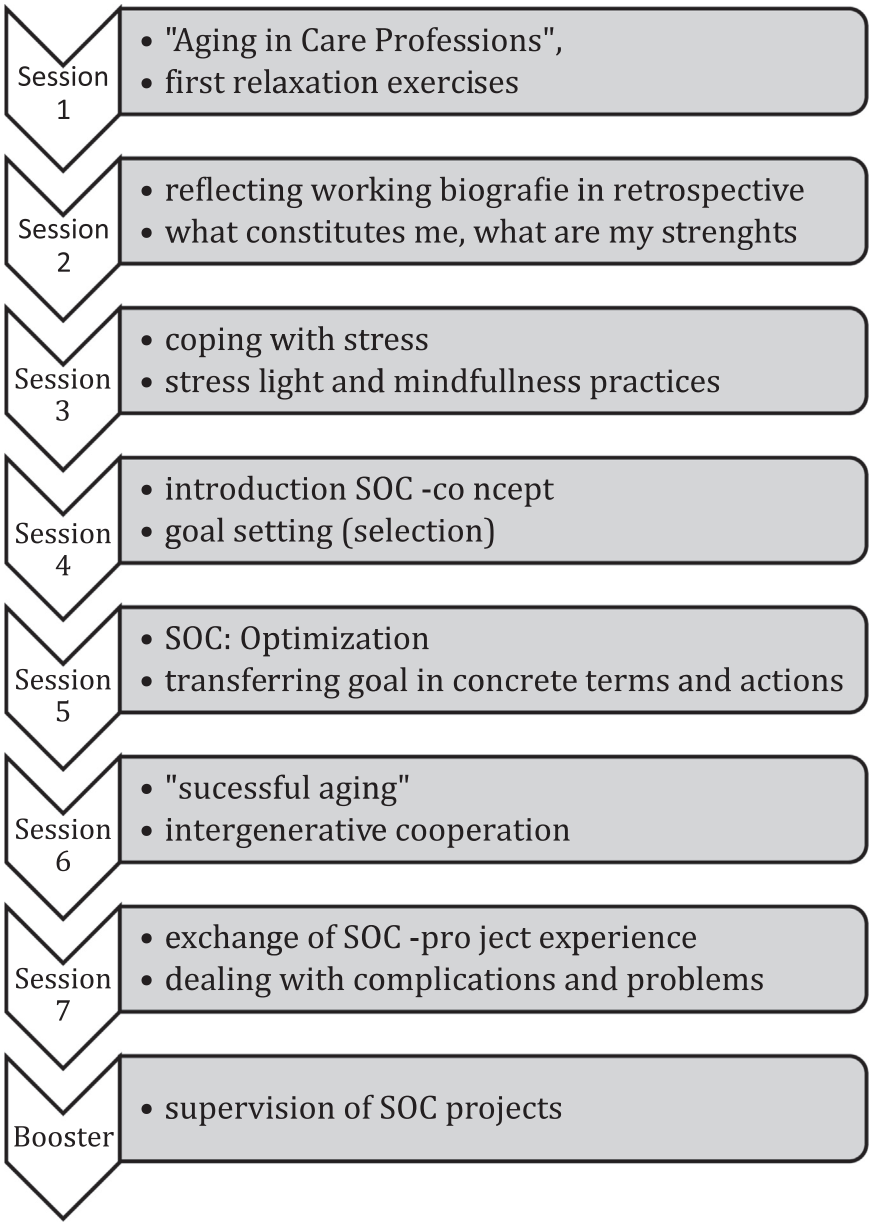

Fig. 2. Key components of the prevention program.

exercises at home with the provided CD. Following insights gained from the pilot trial, the intervention's time frame was modified from a ten week program with sessions lasting 90 min into a seven week program with sessions lasting 120 min each. The key components of the individual sessions of the final intervention are shown in Fig. 2.

The first session focused on aging *in care professions.* Apart from getting to know each other as a group, the discussion centered on what aging in care professions meant to participants and which difficulties they encountered and resources they saw. Biographical work was the theme of the second session. Reflections on job choice as well as preceding influences were made. Furthermore, the group sought to identify job history-related patterns and dogmas. In the third session participants elucidated individual stress patterns and reactions and developed stress matrices and possible strategies to avoid them. SOC theory, individual goal setting, and the reflection of urgent fields for action were the main points in session four. Session five focused on optimization: thinking about possible job-related projects and ways to reach the goal. Participants were invited to find, discuss, and reflect first steps of their prevention project in small groups promoting individual collegial advice. In Session seven, age stereotypes as well as the progression of the individual prevention projects were discussed. The focus of the discussion lay on successful aging, stereotypes and working together with different age groups as well as dealing with generation conflicts. In a final booster session, progress of the prevention projects was reported and possible conclusions voiced.

6. Phase 3: definitive RCT

The definitive RCT is being carried out at 3 trial sites in Germany: the Medical Hospitals of the University of Heidelberg, Düsseldorf, and Ulm. At all trial sites, participants were recruited in the accordance with staff councils and nursing management. The RCT's primary objective is to evaluate the effect of the 7 weeks prevention program in terms of quality of life, well-being,

psychological stress, and workability compared to a control (waiting-list) group. In addition, the cost of our intervention will be determined. The monetary value of our intervention will be associated to the primary outcomes in the study to calculate the primary health economy measure "Incremental Cost-effectiveness Ratio" (ICER). The ICER will provide in monetary terms the additional cost of the respective study intervention to be paid for gaining an additional outcome. The specific stochastic uncertainty of the ICER will be addressed by applying bootstrapping procedures, calculating cost-effectiveness acceptability curves (CEACs), willingness-to-pay criteria and sensitivity analyses etc (Salize & Kilian, 2010). For additional health economy findings, a series of cost-utility analyses will be conducted. Here, the quality of life of study participants will be set as a primary outcome. Quality of life data, as assessed with the WHO-Qol-Bref (WHO 1996), will be transformed to quality-adjusted life years (QALYs) that are gained or lost over time due to the respective trial intervention. The QUALY-gain will be rated against the intervention cost (Salize & Kilian, 2010). Accordingly, the study is registered at a primary clinical trial registry (ISRCTN). The study protocol will be published separately.

7. Discussion

Nursing envelops many psychological and physiological work-related stresses (Bernal et al., 2015). Research has shown that levels of work-related psychological distress are high in healthcare workers (Iacovides, Fountoulakis, Kaprinis, & Kaprinis, 2003). Health and pension insurance statistics indicate high sick leave rates among nurses due to mental health-related diagnoses (Isfort et al., 2010; McVicar, 2003; Ruotsalainen et al., 2015). In order to deal with rising (mental health-related) problems of employees in the health care sector, we developed an intervention that targeted the high risk group of older nursing staff. In the presented article, we have described the developmental steps of our preventive intervention for nurses aged 45 years and older in detail. Prior to the definitive RCT with the objective of investigating effectiveness, we aimed to explore the main sources of workplace distress and how they may be targeted. Additionally, a qualitative study with nursing managers and ward management as well as experiences gained in our pilot study informed about barriers and beneficial factors in the implementation of this intervention program. The phased approach to the development and evaluation of complex interventions proposed by the medical research council proved to be an auxiliary tool for the design of our intervention (Campbell et al., 2000). We could show that a complex intervention program can be developed and implemented for nursing staff aged 45, reach the targeted population, and gains high acceptance. However, further research on feasibility and effectiveness is indicated. Results of our RCT will be published elsewhere.

References

Afentakis, A., & Maier, T. (2010). *Projektionen des Personalbedarfs und -angebots in Pflegeberufen Bis 2025*. Wiesbaden: Statistisches Bundesamt.

Baltes, P. B. (1997). On the incomplete architecture of human ontogeny. Selection, optimization, and compensation as foundation of developmental theory. *American Psychologist*, *52*(4), 366–380.

Baltes, P. B., & Baltes, M. M. (1990). Psychological perspectives on successful aging: The model of selective optimization with compensation In: P. B. Baltes, & M. M. Baltes (Eds.), *Successful aging: perspectives from the behavioral sciences* (pp. 1–34). New York: Cambridge University Press.

Bernal, D., Campos-Serna, J., Tobias, A., Vargas-Prada, S., Benavides, F. G., & Serra, C. (2015). Work-related psychosocial risk factors and musculoskeletal disorders in hospital nurses and nursing aides: A systematic review and meta-analysis. *International Journal Nursing Studies*, *52*(2), 635–648.

Bohnsack, R., & Przyborski, A. (2006). Diskursorganisation, Gesprächsanalyse und die Methode der Gruppendiskussion In: R. Bohnsack (Ed.), *Das Gruppendiskussionsverfahren in der Forschungspraxis* (pp. 233–248). Opladen: Budrich.

Camerino, D., Conway, P. M., Van der Heijden, B. I., Estryn-Behar, M., Consonni, D., Gould, D., & Hasselhorn, H. M. (2006). Low-perceived work ability, ageing and intention to leave nursing: a comparison among 10 European countries. *Journal Advanced Nursing*, *56*(5), 542–552. http://dx.doi.org/10.1111/j.1365-2648.2006.04046.x.

Campbell, M., Fitzpatrick, R., Haines, A., Kinmonth, A. L., Sandercock, P., Spiegelhalter, D., & Tyrer, P. (2000). Framework for design and evaluation of complex interventions to improve health. *BMJ*, *321*(7262), 694–696.

Carlson, C. R., & Hoyle, R. H. (1993). Efficacy of abbreviated progressive muscle relaxation training: a quantitative review of behavioral medicine research. *Journal Consulting Clinical Psychology*, *61*(6), 1059.

Cloostermans, L., Bekkers, M. B., Uiters, E., & Proper, K. I. (2014). The effectiveness of interventions for ageing workers on (early) retirement, work ability and productivity: a systematic review. *International Archives Occupational Environmental Health*, *88*(5), 521–532.

Coomber, B., & Barriball, K. L. (2007). Impact of job satisfaction components on intent to leave and turnover for hospital-based nurses: a review of the research literature. *International Journal Nursing Studies*, *44*(2), 297–314.

Demerouti, E., Bakker, A. B., Nachreiner, F., & Schaufeli, W. B. (2001). The job demands-resources model of burnout. *Journal Applied Psychology*, *86*(3), 499.

Flick, U., Kardorff, E., & Steinke, I. (2010). *Qualitative forschung: ein handbuch* (8 ed.). Reinbek: Rowohlt-Taschenbuch-Verlag.

Freund, A. M., & Baltes, P. B. (2000). The orchestration of selection, optimization, and compensation: an action-theoretical conceptualization of a theory of developmental regulation In: W. J. Perrig, & A. Grob (Eds.), *Control of human behaviour, mental processes and consciousness* (pp. 35–38). Mahwah, NJ: Erlbaum.

Gan, Y., Yang, C., Tong, X., Sun, H., Cong, Y., Yin, X., & Gong, Y. (2014). Shift work and diabetes mellitus: a meta-analysis of observational studies. *Occupational Environmental Medicine*. (oemed-2014-102150)

Grossman, P., Niemann, L., Schmidt, S., & Walach, H. (2004). Mindfulness-based stress reduction and health benefits: a meta-analysis. *Journal Psychosomatic Research*, *57*(1), 35–43.

Haber, D. (2006). Life review: Implementation, theory, research, and therapy. *The International Journal of Aging and Human Development*, *63*(2), 153–171.

Hasselhorn, H.-M., Tackenberg, P., & Peter, R. (2004). Effort–reward imbalance among nurses in stable countries and in countries in transition. *International Journal Occupational Environmental Health*, *10*(4), 401–408.

Iacovides, A., Fountoulakis, K., Kaprinis, S., & Kaprinis, G. (2003). The relationship between job stress, burnout and clinical depression. *Journal Affective Disorders*, *75*(3), 209–221.

Isfort, M., Weidner, F., Neuhaus, A., Kraus, A., Köster, V.-H., & Gehlen, D. (2010). Pflegethermometer 2009. Eine bundesweite Befragung von Pflegekräften zur Situation der Pflege und Patientenversorgung im Krankenhaus. *Köln: German Institute Applied Nursing Research*.

Johnson, J. E., Burlingame, G. M., Olsen, J. A., Davies, D. R., & Gleave, R. L. (2005). Group climate, cohesion, alliance, and empathy in group psychotherapy: multilevel structural equation models. *Journal Counseling Psychology*, *52*(3), 310.

Kaluza, G. (2012). *Gelassen und sicher im stress: das stresskompetenz-buch-stress erkennen, verstehen*. Bewältigen: Springer-Verlag.

Lambert, M. J. (2013). *Bergin and Garfield's handbook of psychotherapy and behavior change*. John Wiley & Sons.

Limm, H., Gundel, H., Heinmuller, M., Marten-Mittag, B., Nater, U. M., Siegrist, J., & Angerer, P. (2011). Stress management interventions in the workplace improve stress reactivity: a randomised controlled trial. *Occupational Environmental Medicine*, *68*(2), 126–133. http://dx.doi.org/10.1136/oem.2009.054148.

Mayring, P. (2008). *Qualitative inhaltsanalyse: grundlagen und techniken* (10 ed.). Weinheim, Basel: Beltz.

McVicar, A. (2003). Workplace stress in nursing: a literature review. *Journal Advanced Nursing*, *44*(6), 633–642.

Müller, A., Heiden, B., Herbig, B., Poppe, F., & Angerer, P. (2015). Improving well-being at work: a randomized controlled intervention based on selection, optimization, and compensation. *Journal Occupational Health Psychology*.

Müller, A., & Weigl, M. (2015). Selection, optimization, and compensation at work in relation to age. *Encyclopedia Geropsychology*.

Muller, A., Weigl, M., Heiden, B., Glaser, J., & Angerer, P. (2012). Promoting work ability and well-being in hospital nursing: the interplay of age, job control, and successful ageing strategies. *Work*, *41*(1), 5137–5144. http://dx.doi.org/10.3233/wor-2012-0083-5137.

Müller, A., Weigl, M., Heiden, B., Herbig, B., Glaser, J., & Angerer, P. (2013). Selection, optimization, and compensation in nursing: exploration of job-specific strategies, scale development, and age-specific associations to work ability. *Journal Advanced Nursing*, *69*(7), 1630–1642. http://dx.doi.org/10.1111/jan.12026.

Ng, R., Allore, H. G., Trentalange, M., Monin, J. K., & Levy, B. R. (2015). Increasing negativity of age stereotypes across 200 years: evidence from a database of 400 million words. *PloS One*, *10*(2), e0117086.

Reddemann, L. (2010). *Imagination als heilsame Kraft. Zur Behandlung von Traumafolgen mit ressourcenorientierten Verfahren*. 141. Germany: Klett-Cotta.

Richardson, K. M., & Rothstein, H. R. (2008). Effects of occupational stress management intervention programs: a meta-analysis. *Journal Occupational Health Psychology*, *13*(1), 69–93.

Rothermund, K., & Brandtstädter, J. (2003). Age stereotypes and self-views in later life: Evaluating rival assumptions. *International Journal Behavioral Development*, *27*(6), 549–554.

Ruotsalainen, J. H., Verbeek, J. H., Marine, A., & Serra, C. (2015). Preventing

occupational stress in healthcare workers. *Cochrane Database Systematic Reviews*, *4*, CD002892. http://dx.doi.org/10.1002/14651858.CD002892.pub5.
Salize, H., & Kilian, R. (2010). *Gesundheitsökonomie in der psychiatrie. Konzepte, methoden, analysen*. Stuttgart: Kohlhammer.
Schultz, J. H., & Luthe, W. (1959). Autogenic training: a psychophysiologic approach to psychotherapy. *Journal Nervous Mental Disease*.
Zacher, H., & Frese, M. (2011). Maintaining a focus on opportunities at work: The interplay between age, job complexity, and the use of selection, optimization, and compensation strategies. *Journal Organizational Behavior*, *32*(2), 291–318.
