# Supplementary material for: Healthy ageing at work— Efficacy of group interventions on the mental health of nurses aged 45 and older: Results of a randomised, controlled trial
Source: PLoS One. 2018 Jan 19;13(1):e0191000. doi: 10.1371/journal.pone.0191000 (PMC5774702; doi:10.1371/journal.pone.0191000)
Supplement: S2 File — (PDF) [file pone.0191000.s002.pdf]

Version 1_19.12.2013

Development and Evaluation of an Intervention to preserve health and workability and nurses during the life span

- Study protocol -

Leader of the study: Dr. med. Imad Maatouk

Responsible study doctor: Dr. med. Imad Maatouk

Tel: 06221-56-37585
Email. Imad.Maatouk@med.uni-heidelberg.de

Department of General Internal Medicine and Psychosomatics,
University Hospital of Heidelberg
Thibautstraße 2,
69115 Heidelberg

Other participating scientists:

- Prof. Dr. med. Wolfgang Herzog, Dipl.-Psych. Kirsten Herbst
- Klinik für Allgemeine Medizin und Psychosomatik, Universitätsklinikum Heidelberg
- Prof. Dr. med .Peter Angerer und Dr. Andreas Müller, Institut für Arbeitsmedizin und Sozialmedizin, Universitätsklinikum Düsseldorf, Universitätsstraße 5, 40225 Düsseldorf
- Prof. Dr. med. Harald Gündel, Universitätsklinik für Psychosomatische Medizin und Psychotherapie, Albert-Einstein-Allee 23, 89081 Ulm

Application for research grant: Kompetenzzentrums Prävention psychischer und psychosomatischer Erkrankungen in der Arbeits- und Ausbildungswelt (Ministerium für Wissenschaft, Forschung und Kunst, Baden-Württemberg) – waiting for approval

As a result of demographic change nursing shortage and changes within the composition of staffs in the nursing sector are expected. Self-reported workability decreases at the age of 45 years due to psychological and physical strain. Therefore, the aim of this study is to evaluate a preventive group intervention for older nurses.

Before designing the intervention, qualitative interviews and focus groups were performed, to allow to consider the view of the target population in the development process. Based on a needs assessment and theory, the invervention was created.

Together with the staff during the intervention concepts for labour organization of an ageing workforce will be created and applied und and personal ressources are strengthened, to better deal with strain and to preserve health.

As a result, the nursing department will obtain an evidence based intervention to preserve health of healthcare workers.

One important theory of successful ageing will be applied. The theory of Selection (focusing on fewer important goals), Optimization (utilise resources in an effective manner) and Compensation (the use of adaptive aids to preserve performance) can help to preserve health and performance in old age.

Due to the setting of small groups the intervention will be inexpensive.

TOC

1. Introduction 4
2. Aims of the study 4
3. Aims 5
4. Test procedere 5
5. Study design 8
6. Inclusion Criteria 8
7. Exclusion criteria 9
8. Process of the study 9
9. Abort criteria 10
10. Statistical design 10
    Sample size estimation 10
    Statistical analyses 10
11. Ethical principles and legal bases 10
12. Signature 11
13. Literature 12

1. Introduction

Demographic change leads to shortfalls in nursing numbers. According to projections by German Statistics office these changes will entail a shortage of more than 112.000 full time nurses(1). In addition, nursing is associated with high physical and psychological strain. To deal with them, many skills such as physical strength, cognitive skills or adaption to desynchronization are necessary. Those skills diminish in older age. Therefore, self-reported workability declines at the age of 45 years. Further, low workability is linked to a high intention to leave the job (2).

To preserve the work force of nurses, it is important to develop preventive intervention to support nurses in preserving health and performance status.

Conceptual foci will be set on ageing at work, stress prevention, reflecting the working biography (10), Health, Work-Life Balance, strengthen personal resources and coping strategies and supportive group intervention (8).

One important theory of successful ageing will be applied. The theory of Selection (focusing on fewer important goals), Optimization (utilize resources in an effective manner) and Compensation (the use of adaptive aids to preserve performance) can help to preserve health and performance in old age. There is a large body of evidence on SOC in the work context (5).

Preliminary work was performed at the university hospital of Düsseldorf. Knowledge about SOC in the work place could be expanded. Results suggest, that a) beyond general strategies of SOC nurses use context-specific strategies of SOC (4) and b) SOC can be helpful if working environment is favorable (4). Therefore, the working environment should be designed in a manner that allows the application of SOC-strategies. An intervention that aims to enable the application of SOC has to consider the individual and the organizational level of preventive strategies (6).

Interventional strategies that are known to be effective (10) with regard to coping with stress, health, work-wife balance, work-life balance, strengthen personal resources and coping strategies and supportive group intervention are considered (35).

2. Aims of the study

For the prevention of nursing shortfalls, it is important, to develop a working environment, enabling nurses to remain on the job until retirement age.

One strategy to develop the working environment could be the application of the theory of SOC (3,4): The theory of Selection (focusing on fewer important goals), Optimization (utilize resources in an effective manner) and Compensation (the use of adaptive aids to preserve performance) has shown to be helpful in preserving health and performance in old age. The

main aim of the study is to test a group intervention for nurses, aged 45 and older that aims to preserve workability of ageing nurses. Compared to other interventions such as stress prevention (7), individual needs, resources and motivation to apply well-tried strategies shall be considered.

Another goal is the development of personal health competencies. The application of such competencies requires a good working environment (8).

3. Aims

One aim ist he application of SOC strategies and stress prevention strategies (such as relaxation, mindfulness) a good Work-Life Balance while profiting from personal resources to preserve health and workability of nurses. Another aim is the development of an age-based working environment.

The implementation of the intervention „healthy ageing at work“ will…

H01: … have a significant effect on psychological wellbeing/quality of life

H02: … have a significant effect on physical wellbeing/quality of life

H03: … have a significant effect on workability

H04: … have a significant effect on preservation of incapability to work

Secondary aims: The implementation of the intervention „healthy ageing at work“ will…

H05: … have significant effects on self efficacy

H06: … have significant effects on working conditions in terms of job control

H07: … have significant effects on learning conditions

The program contains 10 weekly sessions and a booster-session, each of 90 Minutes. Nurses aged ≥45 are teached in strategies to preserve their health. Sources of strain will be identified and specific

4. Test procedere

General description

Comparison between participants and non-participants (control) with regard to strain. To assess wether nurses benefit from stress prevention programs.

Therapeutic effect

Prior studies have shown high job strain in nurses [9] and an augmented psychological strain in higher age [9].

There is already some evidence about the effectiveness of the application of SOC in another Setting without the supplement of age-related, stress reducing, resource-strengthening modules (such as Work life balance, body, health).

There can be expected, that elderly workers suffer from mental strain. Further, they have few coping strategies with regard to the working environment.

There can be expected that elderly workers with high mental strain benefit from interventions.

Resilience will be promoted, stress can be reduced. Further resource-strengthening with regard to ageing, self-efficacy are also supported by the intervention. Individual projects are developed and implemented at the workplace

Undesirable effects, risk for adverse events

From our point of view there are no risks. – no negative therapeutic effects or undesirable effects are expected. Several validated questionnaires that have been applied in several studies will be used (e.g. [22], [23], [24]). Assessment could even have a relieving (positive) effect, as the assessment is linked to a helpful intervention. If any questions arise or there´s a need for a therapeutic contact, our Department of Psychosomatic Medicine including the emergency service, is available 24/7. Participants will be informed about that. The only strain that could be caused by the intervention is the investment of time, as the sessions are held weekly.

Measures

Psychometric testing

Questionnaires before starting the intervention and 2 other measurement time points will be used. The respective questionnaire is handed out to participants.

Sociodemographic data (age, gender, nationality etc.) are also assessed with questionnaires, including job-related information (Experience, Specialization, Place and kind of job – e.g. OR, ICU, Ward, Outpatient Clinic).

The WHOQOL-BREFM serves at assessing quality of life. A quality adjusted life year or QALY ist a measure for rating of one year in a lifetime in Relation to health. A QALY with the value of 1 represents a year of good health, a QALY with a value of 0 stands for passing away.

The following aspects of quality of life will be considered: psychological subscale, autonomy, social relations, environment and spirituality. There are five subscales representing intensity, capacity, frequency, rating or importance the respective aspect of quality of life.

The GAD-7 is part of the PHQ-D (Patient Health Questionnaire) for diagnosing anxiety disease [11]. It comprises 7 items with 4 possible answers. (0=not at all „3“ nearly every day). The PHQ-D allows monitoring depression [15] and has a good validity [12] [13] [14] and sensitivity of change [18]. In the present study the module for monitoring depression (PHQ-9) will be used.

Work-related self-efficacy is a measure derived from a general self-efficacy scale [18]. The questionnaire measures work related self-confidence. The scale comprises 6 Items; with a 5-Point-Likertscale (1 = not at all; 5 = entirely correct), from which the sum score is calculated. Reliability and Validity are good [18].

The Maslach Burnout Inventory (MBI) allows screening for burnout symptoms – it is the by far most used questionnaire in burn-out research [20]. The German version [21] comprises 22 Items, with a 7-step Likert-scale between 0 (never) and 6 (every day). Die Items there are three subscales: emotional exhaustion (nine Items), depersonalization (five Items), reduced performance (8 Items). For every subscale means are calculated [20]. The MBI showed good validity and reliability [21].

Wellbeing (physical and psychological) and Workability: Psychological Wellbeing will be measured by the WHO-5 [22]. The questionnaire „Irritation“ assesses psychological aspects, that are influenced by work strain [25]. Physical wellbeing will be assessed by the nordic questionnaire [26] which is valid in assessing musculoskeletal problems [27]. The short version of the Work Ability Index (WAI, German version from [28] [29]) will be used.

SOC will be measured by the SOC-questionnaire of Baltes, Baltes, Freund and Lang (1999; [30].) eingesetzt werden. Further a nurse-specific SOC questionnaire will be applied (Müller et al., in preparation) (36).

5. Study design

Study design (with waiting control-group) (Figure 1).

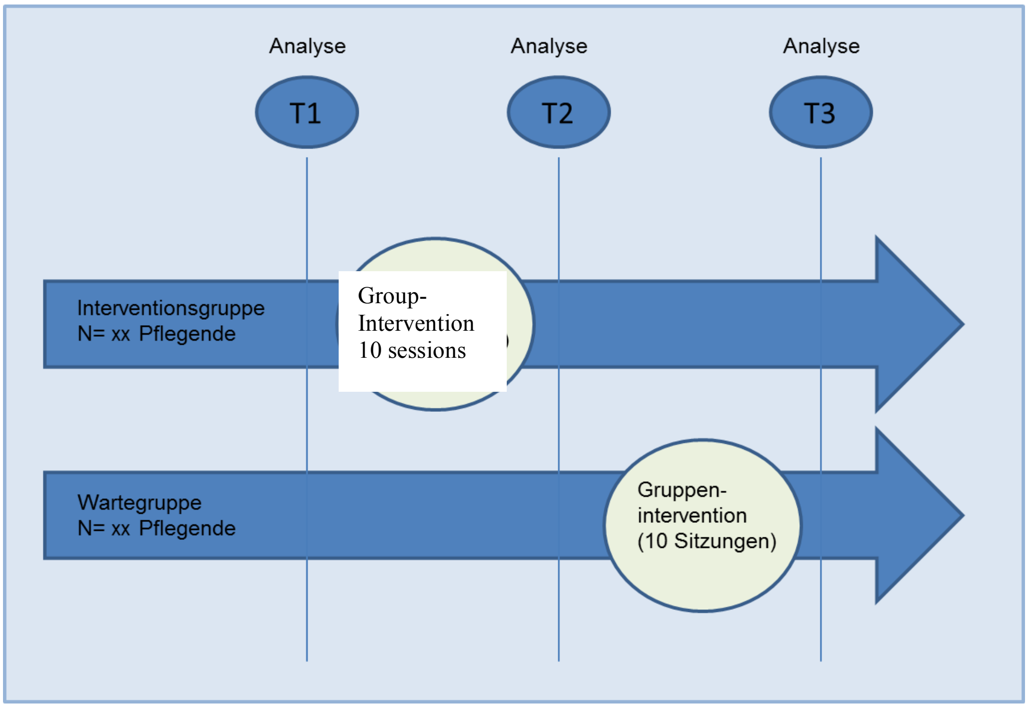

Figure 1: **Waitinglist-Interventiongroup-Design for development and evaluation of the group intervention "ageing at work"**

After written Informed consent has been obtained from the participants, baseline-measurements will be performed. The intervention group will attend a 10-week group intervention „Ageing at work" including a booster-session after 4 weeks. At T2 (14 weeks from T1) new analyses of CG und der IG will be performed. Then, the CG will attend the intervention. CG and IG will be measured again 28 weeks after T1. The waitinglist-controlgroup design ensures that all participants profit from the participation/intervention.

Time-point of measurements were chosen in accordance with to similar evalutation studies that could show positive effects on wellbeing in occupational health related studies and studies that consider individual and organizational aspects. [31] ([32].

6. Inclusion Criteria

Inclusion criteria are

a) working with patients in the nursing sector, Age 45 and older

or b) no leading position that includes workers that belong to a) (lead nurses, nurse manager)

and (for all participants), written informed consent, sufficient knowledge of the German language. The intervention shall be offered to all nurses in the respective age-range to avoid any stigmatization. A Screening with inclusion of high risk would lead to a stigmatization of these people that should be avoided.

7. Exclusion criteria

Exclusion criteria are disability with permantent inability to work or long-lasting inability to work, cognitive impairment or severe physical and mental diseases or emotional strain that does not allow participation to the study.

8. Process of the study

Results of a preliminary qualitative study that aimed to assess the needs and expectations of future participants are considered to design the intervention.
After approval by the nursing management and the staff council, participants will be informed by a letter with general Information about the study. All nurses in the respective age-group at the university hospitals of Heidelberg, Ulm and Düsseldorf will be asked to participate.
After assigning for participation questionnaires will be delivered and filled out on a voluntary basis. Besides the determination on the mentioned concepts with support strategies, there will be a continuous consideration of the participants needs in a flexible way. Composition and content of the group intervention are matched in a chonological manner and are based on a comprehensive conception considering the participants needs.

The intervention comprises: 10 weekly sessions, lasting 90 minutes. The intervention is based on theme-centered interaction. Single sessions deal with wie ageing at the workplace, reflection of the working biography and naming of personal resources, dealing with stressors, the concept of SOC, working on an individual project based on SOC, physical health and work-life-balance. Further several coping strategies will be taught: relaxation excercises/strategies (Mindfulness-based relaxation, Imagination techniques, progressive muscle relaxation).

9. Abort criteria

Participants are allowed to stop participation at any time without giving any reason by withdrawing the declaration of informed consent. Abort of participation is possible at any time if the employee is no longer willing to participate in the study.

10. Statistical design

To eliminate different kinds of bias, the following methods will be applied:
- *Study registration*
- Randomisation by a validated randomisation program
- Standardization of the intervention
- Training and supervision of group leaders
- Blinding of person that is responsible for data acquisition and management

Sample size estimation

Effect size of the main outcome to detect clinical relevance should be 0.5. To estimate sample size we consider similar studies that were implemented in the working and organizational context (33). A sample size of 64 participants has a 80 percent Power do detect such an effect based on a t-test for independent samples with a significance of 0.05 (two sided). Assuming a drop out of 10% we need to include 141 participants in our RCT.

Statistical analyses

Analyses follow the „intention to treat"-principle. Primary outcomes will be analyzed by analysis of covariance. Quality of life and depression at baseline will be considered as covariate in the model respectively. Continuous Outcome-Variables will be analyses by analyses of variance. Dichotomous Outcome-Variables will be tested by Chi-Quadrat-Test or logistic Regression.

11. Ethical principles and legal bases

Ethical principles

The investigation will be performed in accordance with the declaration of Helsinki and the professional code for physicians of the chamber of physicians of Baden-Württemberg in the current version. Participation is voluntary. Declaration of consent can be withdrawn at

any time of the study, without giving any reasons and without penalty/disadvantage. Die Participants will be informed by verbal and written information about the consequences of the investigation, especially about the possible benefit for their health status and potential risks. Approval will be documented by a signature on a written informed consent form. In case of withdrawal data will be destructed or the participant will be asked if he is allowing the evaluation of his data.

Legal bases

The study protocol is sent to the ethics commitee of the der **medical faculty of heidelberg for** evaluation. Recruitment will not start, until the written approval of the ethics commitee is available. Names of the participants and all other confidential information will be used according to the Federal data protection act (BDSG). A transfer of data will only be performed in in a pseudonymous version. Third parties will not be allowed to access original medical files.

12. Signature

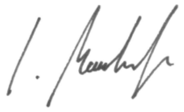

____

Dr. med. Imad Maatouk, Physician

13. Literature

1. Afentakis, A. & Maier, T. (2010). Projektionen des Personalbedarfs und –angebots in Pflegeberufen bis 2025. In statistischem Bundesamt (Ed.) , Wirtschaft und Statistik11/2010: (pp.990-1002) Wiesbaden: Statistisches Bundesamt.
2. Camerino, D., et al. (2006). Low perceived work ability, ageing and intention to leave nursing: a comparison among 10 European countries. Journal of advanced nursing 56.5:542-552.
3. Baltes, P. B., & Baltes, M. M. (1990). Psychological perspectives on successful aging: The model of selective optimization with compensation. In P. B. Baltes & M. M. Baltes (Eds.), Successful aging: Perspectives from the behavioral sciences (pp. 1-34). New York: Cambridge University Press.
4. Muller, A., Weigl, M., Heiden, B., Herbig, B., Glaser, J., & Angerer, P. (2012). Selection,optimization, and compensation in nursing: exploration of job-specific strategies, scale Dokument VF-SP01.rtf 13 development, and age-specific associations to work ability. Journal of advanced nursing.
5. Riediger, M., Li, S.-C., & Lindenberger, U. (2006). Selection, Optimization, and Compensation as Developmental Mechanisms of Adaptive Resource Allocation: Review and Preview. In J. E. Birren & K. W. Schaie (Eds.), Handbook of the Psychology of Aging. 6th Edition (pp. 289-314). Amsterdam: Elsevier.
6. Müller, A.Weigl, M, Lampert, B., Glaser, J. & Angerer, P. (2010). Alterns und altersspezifische Effekte von körperlichen Belastungen und Tätigkeitsspielraum auf die Gesundheit von Altenpflegekräften, Zeitschrift für Arbeitswissenschaft, 64, 229-238.
7. Limm, H., Gundel, H., Heinmuller, M., Marten-Mittag, B., Nater, U. M., Siegrist, J., & Angerer, P. (2011). Stress management interventions in the workplace improve stress reactivity: a randomised controlled trial. Occupational and environmental medicine, 68(2),126-133.
8. Luppa, S.G., Seidler, M. ; Becker, T., Stengler, K. (2013): Psychische Gesundheit und Arbeit: Konzepte, Evidenz und Implikationen für Forschung und Praxis. *Der Nervenarzt*, 84(7), Jul, 2013 832-837.
9. Büssing, A., Glaser, J., Höge, Thomas (2004):Psychische und psysische Belastungen in der ambulanten Pflege: Ein Screening zum Arbeits- und Gesundheitsschutz. *Zeitschrift für Arbeits- und Organisationspsychologie, Vol 48 (4)165-180.*
10. Bühler, K. , Eitel, I.(2008). Der Einfluss der Biografie auf die Stimmung und Befindlichkeit. Nervenheilkund. Zeitshrift für interdisziplinäre Fortbildung., Vol 27 (7)620-632.
11. Kroenke, K., Spitzer, R.L., Williams, J.B. & Lowe, B. (2010). The Patient Health Questionnaire Somatic, Anxiety, and Depressive Symptom Scales: a systematic review. *Gen Hosp Psychiatry, 32*(4), 345-359.
12. Lowe, B., Grafe, K., Zipfel, S., Witte, S., Loerch, B. & Herzog, W. (2004). Diagnosing ICD-10 depressive episodes: superior criterion validity of the Patient Health Questionnaire. *Psychother Psychosom, 73*(6), 386-390.
13. Lowe, B., Spitzer, R.L., Grafe, K., Kroenke, K., Quenter, A., Zipfel, S., Buchholz, C., Witte, S. & Herzog, W. (2004). Comparative validity of three screening questionnaires for DSM-IV depressive disorders and physicians' diagnoses. *J Affect Disord, 78*(2), 131-140.
14. Lowe, B., Kroenke, K., Herzog, W. & Grafe, K. (2004). Measuring depression outcome with a brief self-report instrument: sensitivity to change of the Patient Health Questionnaire (PHQ-9). *J Affect Disord, 81*(1), 61-66.
15. Schwarzer, R. & Jerusalem, M. (1999). *Skalen zur Erfassung von Lehrer- und Schülermerkmalen. Dokumentation der psychometrischen Verfahren im Rahmen der Wissenschaftlichen Begleitung des Modellversuchs Selbstwirksame Schulen.* Berlin: Freie Universität Berlin.

16. Bandura, A. (1997). The anatomy of stages of change. *American Journal of Health Promotion, 12*(1), 8-10.
17. Jerusalem, M. (1990). *Persönliche Ressourcen, Vulnerabilität und Streßerleben.* Göttingen: Hogrefe.
18. Schwarzer, R. (1994). Optimistische Kompetenzerwartung: Zur Erfassung einer personellen Bewältigungsressource. *Diagnostica, 40*(2), 105-123.
19. Abele, A.E., Stief, M. & Andrä, M.S. (2000). Zur ökonomischen Erfassung beruflicher Selbstwirksamkeitserwartungen - Neukonstruktion einer BSW-Skala. *Zeitschrift für Arbeits- und Organisationspsychologie, 44*, 145-151.
20. Maslach, C. & Jackson, S.E. (1981). Maslach Burnout Inventory. Research Edition. Palo Alto, CA: Consulting Psychologists Press.
21. Gumz, A., Erices, R., Brähler, E. & Zenger, M. (2013). Faktorstruktur und Gütekriterien der deutschen Übersetzung des Maslach-Burnout-Inventars für Studierende von Schaufeli et al. (MBI-SS). *Psychotherapie, Psychosomatik, Medizinische Psychologie, 63*(2), 77-84.
22. Krieger T, Zimmermann J, Huffziger S, Ubl B, Diener C, Kuehner C, et al. Measuring depression with a well-being index: further evidence for the validity of the WHO Well-Being Index (WHO-5) as a measure of the severity of depression. Journal of affective disorders. 2014; 156:240-4.
23. Ehrenthal, J.C., Lehr, J., Dinger, U., Köhling, J., Horsch, L., Nikendei, C., Schauenburg, H. & Gierk, B. (2013). Der OPD-Strukturfragebogen (OPD-SF): Entwicklung einer Kurzversion und neue Ergebnisse zur Validität. Heidelberg: 64. Arbeitstagung des Deutschen Kollegiums für Psychosomatische Medizin (DKPM).
24. Ehrenthal, J.C., Dinger, U. & Schauenburg, H. (2006). Erste Ergebnisse der Evaluation einer deutschsprachigen Version des Bindungsfragebogens "Experiences in Close Relationships - Revised (ECR-R)". Magdeburg: 57. Arbeitstagung des Deutschen Kollegiums für Psychosomatische Medizin (DKPM).
25. Mohr, G., Rigotti, T., & Müller, A. (2005). Irritation - ein Instrument zur Erfassung psychischer Befindensbeeinträchtigungen im Arbeitskontext. Skalen- und Itemparameter aus 15 Studien.
Zeitschrift für Arbeits- und Organisationspsychologie, 49, 44-48.
26. Kuorinka, I., Jonsson, B., Kilbom, A., Vinterberg, H., Biering-Sörensen, F., Andersson, G., et al.(1987). Standardised Nordic questionnaires for the analysis of musculoskeletal symptoms. Applied Ergonomics, 18, 233-237.
27. Trinkoff, A. M., Lipscomb, J. A., Geiger-Brown, J., & Brady, B. (2002). Musculoskeletal problems of the neck, shoulder, and back and functional consequences in nurses. American Journal Of Industrial Medicine, 41, 170-178.
28. Prumper, Jochen, and Gottfried Richenhagen. (2011) Von der Arbeitsunfahigkeit zum Haus der Arbeitsfähigkeit. Der Work Ability Index und seine Anwendung. Altere Beschaftigte: Zu jung, um alt zu sein. Konzepte–Forschungsergebnisse–Instrumente. Bertelsmann, Bielefeld: 135-146.
29. Hasselhorn, H.-M., & Freude, G. (2007). Der Work Ability Index. Bremerhaven: Wirtschaftsverlag, NW.
30. Baltes, P. B., Baltes, M. M., Freund, A. M., & Lang, F. R. (1999). The measure of selection, optimization, and compensation (SOC) by self-report. Berlin: Max Planck Institute for Human Development.
31. Limm, H., Gündel, H., Heinmüller, M., Marten-Mittag, B., Nater, U. M., Siegrist, J., et al. (2011). Stressmanagement interventions in the workplace improve stress reactivity: a randomised controlled trial. Occupational And Environmental Medicine, 68, 126-133.
32. Weigl, M., Müller, A., Hornung, S., & Angerer, P. (submitted for publication). Age and work ability:Investigating the moderating roles of job control and individual strategies of selection, optimization and compensation.
33. Richardson KM, Rothstein HR (2008) Effects of occupational stress management intervention programs: a meta-analysis. J Occup Health Psychol 13:69–93

34. Angermeyer, C., Kilian, R. & Mathschinger, H. (2002): Deutschsprachige Version der WHO Instrumente zur Erfassung von Lebensqualität WGOQOL_100 und WHOQOL-BREFM. Zeitschrift für Medizinische Psychologie 1/2002 44-48.
35. Schaefert, R.; Kaufmann, C. et al. (2011): Somatoforme Syndrome in der Hausarztpraxis. Manual zur interdisziplinären Gruppentherapie. *Psychotherapeut 2011,* Schwerpunkt Gruppenpsychotherapie.
36. Büssing, A.; Glaser, J.;Höge, T.(2001): Screening psychischer Belastungen in der stationären Krankenpflege - Belastungsscreening TAA-KH-S. *Diagnostika, Vol47, Nr2. S.77-87.*
