# Supplementary material for: Healthy ageing at work— Efficacy of group interventions on the mental health of nurses aged 45 and older: Results of a randomised, controlled trial
Source: PLoS One. 2018 Jan 19;13(1):e0191000. doi: 10.1371/journal.pone.0191000 (PMC5774702; doi:10.1371/journal.pone.0191000)
Supplement: S3 File — (PDF) [file pone.0191000.s003.pdf]

Version 1_19.12.2013

Entwicklung und Evaluation eines Schulungsprogramms zum Erhalt der Gesundheit und Arbeitsfähigkeit von Pflegekräften über die Erwerbslebensspanne

- Studienprotokoll -

Leiter der Studie: Dr. med. Imad Maatouk

verantwortlicher Studienarzt: Dr. med. Imad Maatouk

Tel: 06221-56-37585
Email. Imad.Maatouk@med.uni-heidelberg.de

Klinik für Allgemeine Innere Medizin und Psychosomatik,
Universitätsklinikum Heidelberg
Thibautstraße 2,
69115 Heidelberg

Weitere (an der Studie) Beteiligte:

- Prof. Dr. med. Wolfgang Herzog, Dipl.-Psych. Kirsten Herbst
- Klinik für Allgemeine Medizin und Psychosomatik, Universitätsklinikum Heidelberg
- Prof. Dr. med .Peter Angerer und Dr. Andreas Müller, Institut für Arbeitsmedizin und Sozialmedizin, Universitätsklinikum Düsseldorf, Universitätsstraße 5, 40225 Düsseldorf
- Prof. Dr. med. Harald Gündel, Universitätsklinik für Psychosomatische Medizin und Psychotherapie, Albert-Einstein-Allee 23, 89081 Ulm

Förderungsantrag gestellt im Rahmen des Kompetenzzentrums Prävention psychischer und psychosomatischer Erkrankungen in der Arbeits- und Ausbildungswelt (Ministerium für Wissenschaft, Forschung und Kunst, Baden-Württemberg) – Bewilligung ausstehend

Aufgrund des demografischen Wandels ist in naher Zukunft mit einem deutlichen Mangel an Pflegepersonal und einer Änderung der Altersstruktur von Belegschaften im Bereich der Pflege zu rechnen. Gleichzeitig nimmt die selbstberichtete Arbeitsfähigkeit von Pflegekräften im Zuge der hohen psychischen und physischen Belastung des Pflegeberufs mit etwa 45 Jahren ab. Ziel ist daher, eine präventive Gruppenintervention zur Förderung des Erhalts der Arbeitsfähigkeit von Pflegekräften im fortgeschrittenen Erwerbsleben zu evaluieren.

Hierzu wurde im Vorfeld eine qualitative Befragung in Form von problemzentrierten Interviews und Fokusgruppen mit der Zielgruppe durchgeführt, um die Perspektive der Betroffenen konsequent bei der Entwicklung der Intervention einzubeziehen. Basierend auf den Vorbefragungen (Needs Assessment) und bewährter theoretischer Konstrukte wurde eine Intervention konzipiert.

Gemeinsam mit der Pflege werden im Rahmen der Intervention alternsgerechte Arbeitsgestaltungsmaßnahmen erarbeitet und umgesetzt und Ressourcen erlernt, um mit den aktuellen Belastungen besser umgehen zu können und die eigene Gesundheit zu erhalten.

Im Ergebnis des Vorhabens steht Einrichtungen der Krankenpflege ein wissenschaftlich fundiertes und praktisch erprobtes Interventionsprogramm zur Verfügung, mit dem die Gesundheit und Arbeitsfähigkeit von den Pflegenden über die Erwerbsspanne hinweg erhalten und gefördert werden könnte.

Vor dem Hintergrund der sich verschärfenden Situation am Arbeitsmarkt ist die eng am individuellen Bedarf der Mitarbeiter und des spezifischen Arbeitsbereiches orientierte Konzeption und Durchführung eines solchen Ansatzes notwendig. Hierbei wird auf eine bewährte Strategie -das SOK Konzept- zurückgegriffen. Es handelt sich dabei um eine Theorie für eine alternsgerechte Arbeitsgestaltung, die aus Selektion (Priorisierung persönlich bedeutsamer Handlungsbereiche), Optimierung (Erwerb und Einsatz von Mitteln zur Aufrechterhaltung des Funktions- und Leistungsniveaus) und Kompensation (Ausgleich von Funktions- und Leistungseinbußen) besteht. Dieses Vorgehen kann maßgeblich zur Gesundheit und Leistungsfähigkeit im Alter beitragen.

Aufgrund des Gruppenansatzes wird es zudem kostengünstig im Rahmen des betrieblichen Gesundheitsmanagements (BGM) umsetzbar sein.

Inhaltsverzeichnis

1. Einleitung/Wissenschaftliche Grundlagen 4
2. Ziele der Studie 5
3. Zielkriterien 5
4. Prüfverfahren 6
5. Studiendesign 9
6. Einschlusskriterien 10
7. Ausschlusskriterien 11
8. Studienablauf 11
9. Abbruchkriterien 12
10. Statistisches Design 12
    Fallzahlplanung 12
    Statistische Analysen 12
11. Ethische und rechtliche Grundlagen 13
12. Unterschrift 13
13. Literatur 14

1. Einleitung/Wissenschaftliche Grundlagen

Aufgrund des demografischen Wandels ist in naher Zukunft mit einem massiven Mangel an Pflegepersonal zu rechnen: Bis zum Jahre 2021 werden nach Berechnungen des Statistischen Bundesamtes in Deutschland 112.000 Pflegevollzeitkräfte fehlen (1). Erschwerend kommt hinzu, dass der Pflegeberuf eine Tätigkeit mit hohen psychischen und physischen Belastungen ist. Zur Bewältigung sind u.a. eine Reihe solcher Fähigkeiten wie körperliche Kräfte, bestimmte kognitive Fähigkeiten (z.B. Merkfähigkeit) oder die Anpassung an eine Desynkronisation innerer und äußerer Zeitgeber bei Schichtarbeit erforderlich, die im allgemeinen mit dem höheren Lebensalter abnehmen. Dementsprechend nimmt die selbstberichtete Arbeitsfähigkeit von Pflegekräften mit etwa 45 Jahren ab. Verminderte Arbeitsfähigkeit steht wiederum im Zusammenhang mit der Absicht, den Pflegeberuf zu verlassen (2).

Damit Pflege in Zukunft sichergestellt werden kann, ist es demnach von außerordentlicher Bedeutung, Präventionsprogramme zu entwickeln, die ältere Pflegekräfte unterstützen, bis ins hohe Erwerbsalter gesund und leistungsfähig zu bleiben.

Es werden dabei Konzepte berücksichtigt, deren Wirksamkeit empirisch in unterschiedlichen Kontexten überprüft wurde. Foki liegen dabei auf den Bereichen Älter werden im Beruf, Umgang mit Stressoren, Auseinandersetzung mit der eigenen Biografie (10), Stressbewältigung, Gesundheit, Work-Life Balance, Erlernen von Ressourcen und Bewältigungsstrategien und supportiven Gruppentherapieverfahren (8).

Ein Ansatzpunkt für eine alternsgerechte Arbeitsgestaltung ist die Theorie der Selektion, Optimierung, Kompensation (SOK) (3): Die empirische Befundlage zeigt, dass Handlungsstrategien im Sinne von Selektion (Priorisierung persönlich bedeutsamer Handlungsbereiche), Optimierung (Erwerb und Einsatz von Mitteln zur Aufrechterhaltung des Funktions- und Leistungsniveaus), Kompensation (Ausgleich von Funktions- und Leistungseinbußen) maßgeblich zu Gesundheit und Leistungsfähigkeit im Alter beitragen können (5):

Vorarbeiten wurden vor allem am Universitätsklinikum in Düsseldorf durchgeführt. Dort konnte das vorhandene Wissen zu SOK im Arbeitskontext um neue Erkenntnisse erweitert werden. Diese Ergebnisse weisen erstmals darauf hin, dass a) Pflegende neben den bekannten globalen SOK-Strategien auch tätigkeitsspezifische SOK-Strategien zum Erhalt ihrer Gesundheit und Arbeitsfähigkeit nutzen (4) und dass b) SOK-Strategien insbesondere dann die Arbeitsfähigkeit von älteren Pflegenden fördern können, wenn Tätigkeitsbedingungen günstig gestaltet sind (4). In der Konsequenz bedeutet dies, dass nicht nur die einzelnen Pflegenden SOK-Strategien kennen und beherrschen müssen, sondern dass Arbeitsbedingungen so gestaltet sein sollten, dass die Anwendung dieser Strategien auch praktisch möglich ist. Somit muss eine Intervention zur Verstärkung von

SOK-Strategien in der Pflege zwingend an beiden Ebenen - Verhalten und Verhältnisse - gemeinsam ansetzen. (6).
Interventionskonzepte, die sich im Rahmen von Stresspräventionsprogrammen sind wirksam im Hinblick auf Umgang mit Stressoren, Auseinandersetzung mit der eigenen Biografie (10), Stressbewältigung, Gesundheit, Work-Life Balance, Erlernen von Ressourcen und Bewältigungsstrategien und supportiven Gruppentherapieverfahren (35).

2. Ziele der Studie

Damit Pflege in Zukunft sichergestellt werden kann, ist es von außerordentlicher Bedeutung, Arbeitsbedingungen so zu gestalten, dass Pflegekräfte bis ins hohe Erwerbsalter im Pflegeberuf verbleiben können und wollen.
Ein zentraler Ansatzpunkt für eine alternsgerechte Arbeitsgestaltung ist die Theorie der Selektion, Optimierung, Kompensation (3,4): Die empirische Befundlage zeigt, dass Handlungsstrategien im Sinne von Selektion (Priorisierung persönlich bedeutsamer Handlungsbereiche), Optimierung (Erwerb und Einsatz von Mitteln zur Aufrechterhaltung des Funktions- und Leistungsniveaus), Kompensation (Ausgleich von Funktions- und Leistungseinbußen) maßgeblich zu Gesundheit und Leistungsfähigkeit im Alter beitragen können
Ein Hauptziel der vorliegenden Studie ist es, eine neu konzipierte präventive Gruppenintervention zur Erhaltung und Förderung der Arbeitsfähigkeit von Pflegekräften im fortgeschrittenen Erwerbsalter zu erproben. Im Vergleich zu rein (psycho-)edukativen Interventionen und etablierten evidenzbasierten präventiven Interventionen (z.B. zur Stressbewältigung (7)) sollen die individuellen Bedürfnisse der Zielgruppe, ihre vorhandenen Ressourcen und die Motivation zum Einsatz von bewährten Handlungsstrategien größtmögliche Berücksichtigung finden und anhand konkreter Projekte Ziele umgesetzt werden, die eine Verbesserung des Arbeitslebens bzw. eine Reduzierung von belastenden Faktoren mit sich bringt. Neben der Erarbeitung und praktischen Umsetzung von SOK als Strategie erfolgreichen Alterns im Beruf, werden weitere
Ein weiteres Ziel der Intervention ist die Entwicklung persönlicher verhaltensbezogener Gesundheitskompetenzen. Voraussetzung für die erfolgreiche Anwendung dieser Kompetenzen ist die Schaffung gesundheitsfördernder Arbeitsbedingungen (8).

3. Zielkriterien

Ziel ist die Erlernung und aktive Anwendung individueller SOK Strategien sowie Stressbewältigungstechniken (wie zum Beispiel Entspannungsverfahren,

Achtsamkeitsübungen) sowie einem gesundheitsbewussten auf Work-Life Balance ausgerichtetem Umgang mit den eigenen Ressourcen sowie Umgang mit dem Alter.
Durch die Intervention sollen persönliche Ressourcen effektiver und alternsgerechter zur Bewältigung von Beruflichen Belastungen eingesetzt und damit die Gesundheit und Arbeitsfähigkeit von Pflegekräften erhalten werden. Ein weiteres Ziel ist es, alternsgerechte Arbeitsbedingungen zu schaffen.
Mit der Durchführung des Schulungs- und Interventionsprogramms „Älter werden im Beruf" wird/werden im Vergleich zur Nicht-Durchführung …
H01: … das psychische Wohlbefinden/Lebensqualität der Pflegenden signifikant verbessert.
H02: … das körperliche Wohlbefinden/Lebensqualität der Pflegenden signifikant verbessert.
H03: … die Arbeitsfähigkeit der Pflegenden signifikant verbessert.
H04: … die AU-Tage der Pflegenden signifikant verringert.

Nebenhypothesen: Mit der Durchführung des Schulungs- und Interventionsprogramms „Älter werden im Beruf" werden im Vergleich zur Nicht-Durchführung …
H05: … die Selbstwirksamkeitserwartungen der Pflegenden signifikant erhöht.
H06: … die Tätigkeitsspielräume bei der Pflegearbeit signifikant erhöht.
H07: … die Lernmöglichkeiten bei der Pflegearbeit signifikant vermehrt.

Das Interventionsprogramm umfasst insgesamt 10 Module plus eine Boostersitzung. Die 10 Module finden im wöchentlichen Turnus innerhalb 90min Gruppensitzungen statt, in denen es darum geht, anhand von Gruppeninterventionen mit Pflegekräften ≥45 zu zeigen, welche Strategien sie zur Bewältigung der Belastungen und zur Förderung ihrer Gesundheit anwenden können. Gleichzeitig sollen mögliche Belastungen identifiziert und anhand von konkreten SOK Projekten angegangen werden.

4. Prüfverfahren

Allgemeine Beschreibungen

Untersucht und geprüft werden soll, ob sich Pflegekräfte nach Teilnahme an den Gruppeninterventionen im Vergleich zu Nichtteilnehmern aus der Pflege hinsichtlich ihres Belastungsprofils unterscheiden. Gleichzeitig soll erfasst werden, inwiefern belastete Pflegekräfte von Programmen zur Stressbewältigung profitieren können.

Therapeutische Wirkungen

Bisherige Studien zeigen, dass Pflegekräfte in besonderem Maße als belastet gelten [9] und dass es mit zunehmenden Alter zu einer kontinuierlichen Zunahme an psychischen Belastungen kommt [9].

Erkenntnisse darüber, wie wirksam das Erlernen von SOK-Strategien ist, liegen bereits vor, jedoch in einem anderen Setting und ohne die Ergänzung altersbezogener, stressreduzierender Ressourcenfördender Zusatzmodule (wie z.B. Work-Life Balance, Körper und Gesundheit)

Es ist prinzipiell zu erwarten, dass ältere Pflegemitarbeiter im hohen Maße Belastungen ausgesetzt sind und bislang nur über wenige oder unzureichende Bewältigungsstrategien verfügen. Die zudem einen konkreten Bezug zum Arbeitsalltag beinhalten.

Es wird daher erwartet, dass insbesondere hoch belastete Pflegekräfte im höheren Alter im hohen Maße von den Gruppeninterventionen profitieren würden.

So wird die Resilienz gestärkt, Stress reduziert, Ressourcen zum Umgang mit dem Älter werden gefördert und das eigene Selbstwirksamkeitserleben gestärkt, dadurch, dass konkrete individuelle kleine Projekte innerhalb des Arbeitsplatzes angegangen werden.

Unerwünschte Wirkungen, sonstige Risiken oder Belastungen

Durch die Studienteilnahme entstehen unserer Einschätzung nach keine Risiken – so werden im Rahmen der Studie ausdrücklich keine negativen therapeutischen Effekte oder andere unerwünschte Wirkungen erwartet. Die im Fragebogen eingesetzten Instrumente wurden bereits in zahlreichen publizierten Studien eingesetzt (siehe z.B. [22], [23], [24]) und wurden in der Vergangenheit durch verschiedene Arbeitsgruppen hinreichend erprobt und validiert. Daneben kann die Erfassung der vorhandenen Belastungsfaktoren von den teilnehmenden Studierenden unserer klinischen Erfahrung nach sogar als entlastend erlebt werden, zumal diese Erhebung mit einem Hilfeangebot verknüpft ist. Sollte es dennoch zu Fragen und dem Wunsch eines therapeutischen Kontaktes kommen, steht die Psychosomatische Ambulanz sowie der Psychosomatische Rufdienst der Universitätsklinik für Allgemeine Innere Medizin und Psychotherapie rund um die Uhr zu Verfügung, worüber die Teilnehmer aufgeklärt werden. Belastungen durch die Gruppenintervention selbst entstehen allenfalls durch die zeitliche Beanspruchung durch die wöchentlich stattfindenden Termine.

Instrumente

psychometrische Testung

Die folgenden Fragebogeninstrumente sollen vor Beginn der Gruppenintervention und 2 weiteren Messzeitpunkten zur Anwendung kommen. Ein entsprechender Fragebogen wird nach Möglichkeit an alle Interessierten ausgehändigt.

Die soziodemographischen Daten (Alter, Geschlecht, Nationalität, Muttersprache, sozioökonomische Verhältnisse, usw.) werden mittels Fragebogen erfasst, erweitert durch berufsbezogene Angaben (Berufserfahrung, Fachrichtung, Informationen zum Einsatzbereich – z.B. OP, Intensivstation, Normalstation, Ambulanz).

Der WHOQOL-BREFM dient der Erfassung der gesundheitsbezogenen Lebensqualität und eignet sich auch zur interkulturellen Vergleichbarkeit. Ein qualitätskorrigiertes Lebensjahr (englisch quality adjusted life year oder QALY) ist eine Kennzahl für die Bewertung eines Lebensjahres in Relation zur Gesundheit. Ein QALY von 1 bedeutet ein Jahr in voller Gesundheit, während ein QALY von 0 einem Versterben entspricht. QALY ist damit ein Nutzwert für ein Leben (-sjahr). Der QALY ist die meistgenutzte Kennzahl in der gesundheitsökonomischen Evaluation.

Hierbei werden Aspekte der Lebensqualität im psychischen Bereich, im Bereich der Unabhängigkeit, der sozialen Beziehungen, der Umweltressourcen und der Spiritualität erfasst. Für die Beantwortung der Fragen liegen fünf verschiedene Skalentypen vor, die je nach abgefragtem Bereich entweder Intensität, Kapazität, Häufigkeit, Bewertung oder Wichtigkeit des jeweiligen Merkmales von Lebensqualität eingeschätzt werden.

Der GAD-7 ist ein Modul des PHQ-D (Patient Health Questionnaire) zur Diagnostik von Angststörungen [11]. Das Instrument umfasst 7 Fragen mit jeweils 4 Antwortmöglichkeiten. (0=überhaupt nicht bis „3“ beinah jeden Tag). Der PHQ-D eignet sich auch zur Detektion und Verlaufskontrolle der Depression [15] und zeigt hierbei eine gute Validität [12] [13] [14] und Veränderungssensitivität [18]. Für das skizzierte Forschungsvorhaben ist der Einsatz des Moduls zur Detektion depressiver Erkrankungen (PHQ-9) vorgesehen.

Bei der Skala zur Erfassung genereller beruflicher Selbstwirksamkeitserwartung (BSW) handelt es sich um eine modifizierte Version der SWE für berufsbezogene Fragestellungen [18]. Sie misst das Zutrauen in die eigenen beruflichen Fähigkeiten, also letztlich das berufliche Selbstvertrauen. Die Skala besteht aus sechs Items; es wird eine 5-Punkt-Likertskala eingesetzt (1 = trifft gar nicht zu; 5 = trifft genau zu), aus der anschließend ein Mittelwert über alle Items berechnet wird. Hinreichende Reliabilität sowie konvergente und diskriminante Validität sind gegeben [18].

Der Maslach Burnout Inventory (MBI) gilt als Standard zur Erfassung von Burnout-Symptomen – es ist das am häufigsten eingesetzte Verfahren in der Burnout-Forschung [20]. Die deutsche Version des MBIs [21] umfasst 22 Items, von denen jedes mittels einer 7-stufigen Likertskala zwischen 0 (nie) und 6 (täglich) angegeben wird. Die Items teilen sich auf drei Subskalen auf: emotionale Erschöpfung (neun Items), Depersonalisierung (fünf Items), reduzierte persönliche Leistungsfähigkeit (acht Items). Für jede Subskala werden Mittelwerte berechnet, die Normwerte für niedriges, mittleres und hohes Burnout wurden von Maslach und Jackson [20] übernommen. Das MBI zeigte in der Vergangenheit hinreichende Reliabilität und faktorielle Validität [21].

Psychisches und körperliches Wohlbefinden, Arbeitsfähigkeit: Psychisches Wohlbefinden wird mit dem WHO-5 gemessen [22]. Mit dem Fragebogen „Irritation" wird ein veränderungssensitiver psychischer Indikator erfasst, welcher durch berufliche Belastungen beeinflusst wird [25]. Als Indikator für körperliches Wohlbefinden wird der Nordische Fragebogen [26] eingesetzt, der vielfach zur validen Erfassung von Muskel-Skelett-Beschwerden im Bereich der Pflegetätigkeit angewendet worden ist [27]. Mit der Kurzversion des Work Ability Index (WAI, deutsche Kurzversion von [28] [29]) wird ein vielfach eingesetzter Fragebogen zur Selbsteinschätzung der Arbeitsfähigkeit verwendet.

Für die Erfassung von Selektion, Optimierung, Kompensation (SOK) soll die Kurzversion des SOK-Fragebogens von Baltes, Baltes, Freund und Lang (1999; [30].) eingesetzt werden. Darüber hinaus wird der Fragebogen zur Erfassung pflegespezifischer SOK-Strategien eingesetzt werden (Müller et al., in preparation)(36).

5. Studiendesign

Das Studien-Design entspricht einem Wartekontroll-Interventionsgruppen-Design (Abbildung 1).

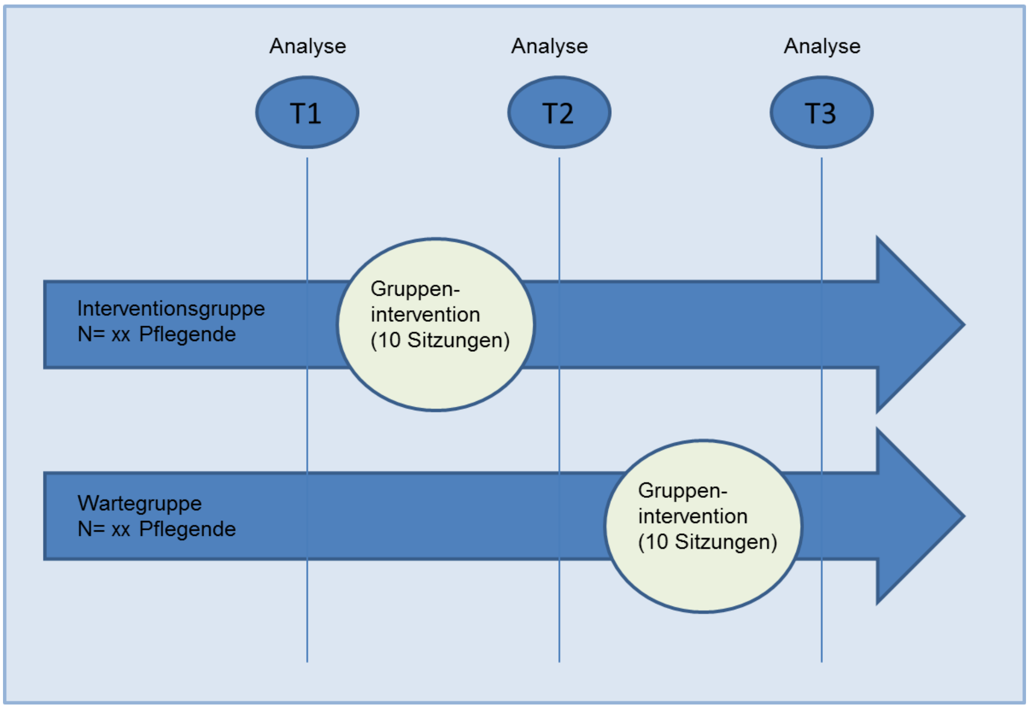

Abbildung 1: **Wartekontroll-Interventionsgruppen-Design zur Entwicklung und Evaluation des Gruppeninterventionsprogramms „Älter werden im Beruf“**

Nach Information und anschließendem schriftlichen Einverständnis der Teilnehmer erfolgt sowohl in der KG als auch in der IG zeitgleich eine Ausgangsanalyse (T1). In der IG wird dann das die 10 wöchige Gruppenintervention „Älter werden im Beruf“ einschließlich der Boostersitzung nach 4 Wochen durchgeführt. Zum Zeitpunkt T2 (14 Wochen nach T1) erfolgt eine erneute Analyse der KG und der IG. Danach erhält die KG die Intervention. In KG und IG erfolgt dann 28 Wochen nach T1 eine erneute Analyse. Mit dem Wartekontroll-Interventionsgruppen-Design wird gewährleistet, dass keiner der Versuchsgruppen die Intervention vorenthalten wird.
Bei der Wahl des Abstandes der Messzeitpunkte beziehen wir uns auf vorhandene Evaluationsstudien zu verhaltens- und verhältnisbezogenen betrieblichen Gesundheitsförderungsmaßnahmen, die positive Effekte auf das Wohlbefinden in vergleichbaren Zeiträumen nachgewiesen haben [31] ([32].

6. Einschlusskriterien

Einschlusskriterien für die Studie sind
a) aktive Ausübung des Pflegeberufs mit direktem Patientenkontakt, Alter ≥ 45 Jahre
oder b) keine aktive Ausübung einer Leitungsfunktion einer Gruppe, die die unter a) bezeichnete Personengruppe einschließt (Stationsleitung, Pflegedienstleitung)

sowie (für alle genannten Gruppen), schriftliche Einverständniserklärung, ausreichende Deutschkenntnisse. Die Intervention soll explizit allen Pflegekräften der genannten Altersgruppe angeboten werden, um eine Stigmatisierung zu vermeiden. Ein Screening mit anschließendem Einschluss einer sogenannten Hochrisikogruppe würde eine Stigmatisierung dieser Personen nach sich ziehen, die vermieden werden soll.

7. Ausschlusskriterien

Ausschlusskriterien sind aktuelle Erwerbsunfähigkeit oder längere Arbeitsunfähigkeit, kognitive Beeinträchtigungen, sowie schwere körperliche und psychiatrische Erkrankungen oder emotionale Belastungen, die eine Studienteilnahme nicht zulassen.

8. Studienablauf

Ergebnisse einer qualitativen Vorstudie zu Bedürfnissen und Erwartungen der potentiellen Teilnehmer fließen in die Gestaltung der einzelnen Gruppensitzungen ein.
Die Teilnehmer werden nach Einholen der Zustimmung der Pflegedienstleitung und des Personalrates im Rahmen eines allgemeinen Anschreibens/Informationsschreibens an alle Pflegekräfte der genannten Altersgruppe des Uniklinikums Heidelberg, sowie der Universitätskliniken Ulm und Düsseldorf über die Gruppenintervention informiert.
Nach der Anmeldung zur Teilnahme werden die Fragebögen zur Verfügung gestellt und auf freiwilliger Basis ausgefüllt. Es erfolgt neben der Festlegung der durch die genannten Konzepte vorgegebenen Unterstützungsstrategien eine kontinuierliches Eingehen auf die Bedürfnisse der Gruppen in Form einer flexiblen Anpassung. Aufbau und Inhalte der Gruppeninterventionen sind jedoch chronologisch aufeinander abgestimmt und liegen einem durchdachten Konzept zugrunde, das auf die Bedürfnisse der Betroffenen eingeht.

Das Interventionsprogramm umfasst:10 Sitzungen a 90 min, die wöchentlich stattfinden. Grundprinzip ist die Themenzentrierte Interaktion. In den einzelnen Sitzungen werden Themenblöcke wie Älter werden im Beruf, Reflektion der Berufsbiografie und Benennung von Ressourcen, Umgang mit Stressoren, SOK-Konzept, persönliche Bearbeitung eines individuellen Projekts im Rahmen von SOK, körperliche Gesundheit und Work-Life-Balance. Darüber hinaus werden den Teilnehmern verschiedene Bewältigungsstrategien in Form von Entspannungsübungen/-techniken (Achtsamkeitsübungen, Phantasiereisen, PMR) vermittelt.

9. Abbruchkriterien

Die Interventionsteilnehmer können die Studie jederzeit ohne Angabe von Gründen durch Widerruf des Einverständnisses abbrechen. Ein Studienabbruch ist jederzeit möglich, wenn der Mitarbeiter nicht mehr bereit ist, an der Studie teilzunehmen.

10. Statistisches Design

Um die verschiedenen Arten von Bias auszuschalten, werden folgende Methoden angewendet:

- *öffentliche Registrierung*
- Randomisierung durch ein validiertes Randomisierungsprogramm
- Standardisierung der Intervention
- Training und Supervision der Gruppenleiter
- Verblindung der Personen, die für Datenerhebung und -auswertung zuständig sind

Fallzahlplanung

Die Effektgröße für das Hauptzielkriterium Lebensqualität sollte bei mind. 0.5 liegen, um eine klinische Relevanz der Intervention nachzuweisen. Bei der Fallzahlplanung beziehen wir uns auf vergleichbare Studien, die im Arbeits- und Organisationsbereich ansetzen (33). Eine Stichprobengröße von 64 Probanden hat eine 80%ige Power um diese Effektgröße mittels eines t-Tests für unabhängige Stichproben bei einem Signifikanzniveau von 0.05 (zweiseitig) zu erhalten. Bei einer angenommenen Drop-Out-Rate von 10% benötigt man mindestens 141 Probanden um sie in den RCT einzuschließen.

Statistische Analysen

Die Analyse des RCT erfolgt nach dem „intention to treat“-Ansatz. Die primären Zielkriterien werden mittels Kovarianzanalyse ausgewertet. Die Lebensqualität bzw. Depressivität bei der Baseline-Erhebung wird dabei als Kovariate im Modell berücksichtigt. Die kontinuierlichen sekundären Outcome-Variablen werden mit Kovarianzanalysen ausgewertet. Dichotome Outcome-Variablen werden mittels Chi-Quadrat-Tests oder logistischer Regression ausgewertet.

11. Ethische und rechtliche Grundlagen

Ethische Grundlagen

Die Untersuchung wird in Übereinstimmung mit der Deklaration von Helsinki und der Berufsordnung für Ärztinnen und Ärzte der Landesärztekammer Baden-Württemberg in den jeweils aktuellen Fassungen durchgeführt. Die Teilnahme an der Untersuchung ist freiwillig. Die Zustimmung kann jederzeit, ohne Angabe von Gründen und ohne Nachteile für die Teilnehmer, zurückgezogen werden. Die Probanden werden vor Studienbeginn schriftlich und mündlich über Wesen und Tragweite der geplanten Untersuchung, insbesondere über den möglichen Nutzen für Ihre Gesundheit und eventuelle Risiken, aufgeklärt. Ihre Zustimmung wird durch Unterschrift auf der Einwilligungserklärung dokumentiert. Bei Rücktritt von der Studie wird bereits gewonnenes (Daten-) Material vernichtet oder beim Probanden angefragt, ob er mit der Auswertung des Materials einverstanden ist.

Rechtliche Grundlagen

Der Studienplan wird vor Studienbeginn der Ethikkommission der **Medizinischen Fakultät Heidelberg** zur Begutachtung vorgelegt. Es wird nicht mit dem Einschluss von Teilnehmern begonnen, bevor nicht die schriftliche, zustimmende Bewertung der Ethikkommission vorliegt. Die Namen der Probanden und alle anderen vertraulichen Informationen unterliegen der ärztlichen Schweigepflicht und den Bestimmungen des Bundesdatenschutzgesetzes (BDSG). Eine Weitergabe von Probandendaten erfolgt ggf. nur in pseudonymisierter Form. Dritte erhalten keinen Einblick in Originalkrankenunterlagen.

12. Unterschrift

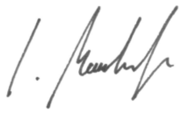

____

Dr. med. Imad Maatouk, Arzt

13. Literatur

1. Afentakis, A. & Maier, T. (2010). Projektionen des Personalbedarfs und –angebots in Pflegeberufen bis 2025. In statistischem Bundesamt (Ed.) , Wirtschaft und Statistik11/2010: (pp.990-1002) Wiesbaden: Statistisches Bundesamt.
2. Camerino, D., et al. (2006). Low perceived work ability, ageing and intention to leave nursing: a comparison among 10 European countries. Journal of advanced nursing 56.5:542-552.
3. Baltes, P. B., & Baltes, M. M. (1990). Psychological perspectives on successful aging: The model of selective optimization with compensation. In P. B. Baltes & M. M. Baltes (Eds.), Successful aging: Perspectives from the behavioral sciences (pp. 1-34). New York: Cambridge University Press.
4. Muller, A., Weigl, M., Heiden, B., Herbig, B., Glaser, J., & Angerer, P. (2012). Selection,optimization, and compensation in nursing: exploration of job-specific strategies, scale Dokument VF-SP01.rtf 13 development, and age-specific associations to work ability. Journal of advanced nursing.
5. Riediger, M., Li, S.-C., & Lindenberger, U. (2006). Selection, Optimization, and Compensation as Developmental Mechanisms of Adaptive Resource Allocation: Review and Preview. In J. E. Birren & K. W. Schaie (Eds.), Handbook of the Psychology of Aging. 6th Edition (pp. 289-314). Amsterdam: Elsevier.
6. Müller, A.Weigl, M, Lampert, B., Glaser, J. & Angerer, P. (2010). Alterns und altersspezifische Effekte von körperlichen Belastungen und Tätigkeitsspielraum auf die Gesundheit von Altenpflegekräften, Zeitschrift für Arbeitswissenschaft, 64, 229-238.
7. Limm, H., Gundel, H., Heinmuller, M., Marten-Mittag, B., Nater, U. M., Siegrist, J., & Angerer, P. (2011). Stress management interventions in the workplace improve stress reactivity: a randomised controlled trial. Occupational and environmental medicine, 68(2),126-133.
8. Luppa, S.G., Seidler, M. ; Becker, T., Stengler, K. (2013): Psychische Gesundheit und Arbeit: Konzepte, Evidenz und Implikationen für Forschung und Praxis. *Der Nervenarzt*, 84(7), Jul, 2013 832-837.
9. Büssing, A., Glaser, J., Höge, Thomas (2004):Psychische und psysische Belastungen in der ambulanten Pflege: Ein Screening zum Arbeits- und Gesundheitsschutz. *Zeitschrift für Arbeits- und Organisationspsychologie, Vol 48 (4)165-180.*
10. Bühler, K. , Eitel, I.(2008). Der Einfluss der Biografie auf die Stimmung und Befindlichkeit. Nervenheilkund. Zeitshrift für interdisziplinäre Fortbildung., Vol 27 (7)620-632.
11. Kroenke, K., Spitzer, R.L., Williams, J.B. & Lowe, B. (2010). The Patient Health Questionnaire Somatic, Anxiety, and Depressive Symptom Scales: a systematic review. *Gen Hosp Psychiatry, 32*(4), 345-359.
12. Lowe, B., Grafe, K., Zipfel, S., Witte, S., Loerch, B. & Herzog, W. (2004). Diagnosing ICD-10 depressive episodes: superior criterion validity of the Patient Health Questionnaire. *Psychother Psychosom, 73*(6), 386-390.
13. Lowe, B., Spitzer, R.L., Grafe, K., Kroenke, K., Quenter, A., Zipfel, S., Buchholz, C., Witte, S. & Herzog, W. (2004). Comparative validity of three screening questionnaires for DSM-IV depressive disorders and physicians' diagnoses. *J Affect Disord, 78*(2), 131-140.
14. Lowe, B., Kroenke, K., Herzog, W. & Grafe, K. (2004). Measuring depression outcome with a brief self-report instrument: sensitivity to change of the Patient Health Questionnaire (PHQ-9). *J Affect Disord, 81*(1), 61-66.
15. Schwarzer, R. & Jerusalem, M. (1999). *Skalen zur Erfassung von Lehrer- und Schülermerkmalen. Dokumentation der psychometrischen Verfahren im Rahmen der Wissenschaftlichen Begleitung des Modellversuchs Selbstwirksame Schulen.* Berlin: Freie Universität Berlin.

16. Bandura, A. (1997). The anatomy of stages of change. *American Journal of Health Promotion, 12*(1), 8-10.
17. Jerusalem, M. (1990). *Persönliche Ressourcen, Vulnerabilität und Streßerleben.* Göttingen: Hogrefe.
18. Schwarzer, R. (1994). Optimistische Kompetenzerwartung: Zur Erfassung einer personellen Bewältigungsressource. *Diagnostica, 40*(2), 105-123.
19. Abele, A.E., Stief, M. & Andrä, M.S. (2000). Zur ökonomischen Erfassung beruflicher Selbstwirksamkeitserwartungen - Neukonstruktion einer BSW-Skala. *Zeitschrift für Arbeits- und Organisationspsychologie, 44*, 145-151.
20. Maslach, C. & Jackson, S.E. (1981). Maslach Burnout Inventory. Research Edition. Palo Alto, CA: Consulting Psychologists Press.
21. Gumz, A., Erices, R., Brähler, E. & Zenger, M. (2013). Faktorstruktur und Gütekriterien der deutschen Übersetzung des Maslach-Burnout-Inventars für Studierende von Schaufeli et al. (MBI-SS). *Psychotherapie, Psychosomatik, Medizinische Psychologie, 63*(2), 77-84.
22. Krieger T, Zimmermann J, Huffziger S, Ubl B, Diener C, Kuehner C, et al. Measuring depression with a well-being index: further evidence for the validity of the WHO Well-Being Index (WHO-5) as a measure of the severity of depression. Journal of affective disorders. 2014; 156:240-4.
23. Ehrenthal, J.C., Lehr, J., Dinger, U., Köhling, J., Horsch, L., Nikendei, C., Schauenburg, H. & Gierk, B. (2013). Der OPD-Strukturfragebogen (OPD-SF): Entwicklung einer Kurzversion und neue Ergebnisse zur Validität. Heidelberg: 64. Arbeitstagung des Deutschen Kollegiums für Psychosomatische Medizin (DKPM).
24. Ehrenthal, J.C., Dinger, U. & Schauenburg, H. (2006). Erste Ergebnisse der Evaluation einer deutschsprachigen Version des Bindungsfragebogens "Experiences in Close Relationships - Revised (ECR-R)". Magdeburg: 57. Arbeitstagung des Deutschen Kollegiums für Psychosomatische Medizin (DKPM).
25. Mohr, G., Rigotti, T., & Müller, A. (2005). Irritation - ein Instrument zur Erfassung psychischer Befindensbeeinträchtigungen im Arbeitskontext. Skalen- und Itemparameter aus 15 Studien.
    Zeitschrift für Arbeits- und Organisationspsychologie, 49, 44-48.
26. Kuorinka, I., Jonsson, B., Kilbom, A., Vinterberg, H., Biering-Sörensen, F., Andersson, G., et al.(1987). Standardised Nordic questionnaires for the analysis of musculoskeletal symptoms. Applied Ergonomics, 18, 233-237.
27. Trinkoff, A. M., Lipscomb, J. A., Geiger-Brown, J., & Brady, B. (2002). Musculoskeletal problems of the neck, shoulder, and back and functional consequences in nurses. American Journal Of Industrial Medicine, 41, 170-178.
28. Prumper, Jochen, and Gottfried Richenhagen. (2011) Von der Arbeitsunfahigkeit zum Haus der Arbeitsfähigkeit. Der Work Ability Index und seine Anwendung. Altere Beschaftigte: Zu jung, um alt zu sein. Konzepte–Forschungsergebnisse–Instrumente. Bertelsmann, Bielefeld: 135-146.
29. Hasselhorn, H.-M., & Freude, G. (2007). Der Work Ability Index. Bremerhaven: Wirtschaftsverlag, NW.
30. Baltes, P. B., Baltes, M. M., Freund, A. M., & Lang, F. R. (1999). The measure of selection, optimization, and compensation (SOC) by self-report. Berlin: Max Planck Institute for Human Development.
31. Limm, H., Gündel, H., Heinmüller, M., Marten-Mittag, B., Nater, U. M., Siegrist, J., et al. (2011). Stressmanagement interventions in the workplace improve stress reactivity: a randomised controlled trial. Occupational And Environmental Medicine, 68, 126-133.
32. Weigl, M., Müller, A., Hornung, S., & Angerer, P. (submitted for publication). Age and work ability:Investigating the moderating roles of job control and individual strategies of selection, optimization and compensation.
33. Richardson KM, Rothstein HR (2008) Effects of occupational stress management intervention programs: a meta-analysis. J Occup Health Psychol 13:69–93

34. Angermeyer, C., Kilian, R. & Mathschinger, H. (2002): Deutschsprachige Version der WHO Instrumente zur Erfassung von Lebensqualität WGOQOL_100 und WHOQOL-BREFM. Zeitschrift für Medizinische Psychologie 1/2002 44-48.
35. Schaefert, R.; Kaufmann, C. et al. (2011): Somatoforme Syndrome in der Hausarztpraxis. Manual zur interdisziplinären Gruppentherapie. *Psychotherapeut 2011,* Schwerpunkt Gruppenpsychotherapie.
36. Büssing, A.; Glaser, J.;Höge, T.(2001): Screening psychischer Belastungen in der stationären Krankenpflege - Belastungsscreening TAA-KH-S. *Diagnostika, Vol47, Nr2. S.77-87.*
